# Design, synthesis and biological evaluation of novel betulinic acid derivatives

**DOI:** 10.1186/1752-153X-6-141

**Published:** 2012-11-23

**Authors:** Shengjie Yang, Na Liang, Hu Li, Wei Xue, Deyu Hu, Linhong Jin, Qi Zhao, Song Yang

**Affiliations:** 1 State Key Laboratory Breeding Base of Green Pesticide and Agricultural Bioengineering, Key Laboratory of Green Pesticide and Agricultural Bioengineering, Ministry of Education, Guizhou University, Guiyang 550025, P.R. China; 2 Ctr for R&D of Fine Chemicals, Guizhou University, Huaxi St, Guiyang 550025, China

## Abstract

**Background:**

Tumor, is one of the major reason for human death, due to its widespread occurrence. Betulinic acid derivatives have attracted considerable attention as cancer chemopreventive agents and also as cancer therapeutics. Many of its derivatives inhibit the growth of human cancer cell lines by triggering apoptosis. With this background, we planned to synthesize a series of betulinic acid derivatives to assess their antiproliferation efficacy on human cancer cell lines.

**Results:**

A series of novel betulinic acid derivatives were designed and synthesized as highlighted by the preliminary antitumor evaluation against MGC-803, PC3, A375, Bcap-37 and A431 human cancer cell lines *in vitro*. The pharmacological results showed that some of the compounds displayed moderate to high levels of antitumor activities with most of new exhibiting higher inhibitory activities compared to BA. The IC_50_ values of compound 3c on the five cancer cell lines were 2.3, 4.6, 3.3, 3.6, and 4.3 μM, respectively. Subsequent fluorescence staining and flow cytometry analysis (FCM) indicated that compound 3c could induce apoptosis in MGC-803 and PC3 cell lines, and the apoptosis ratios reached the peak (37.38% and 33.74%) after 36 h of treatment at 10 μM.

**Conclusions:**

This study suggests that most of betulinic acid derivatives could inhibit the growth of human cancer cell lines. Furthermore, compound 3c could induce apoptosis of cancer cells.

## Background

Malignant neoplasm is the major cause of human death worldwide, mainly due to its high occurrence. Thousands of people die of cancer every year, despite aggressive treatment regimens including surgery, chemotherapy, radiation therapy, and palliative care. The situation demands urgent need for new therapies of therapeutic combinations to improve the survival and quality of life of cancer patients.

Betulinic acid (BA, 3β-3-hydroxy-lup-20(29)-en-28-oic acid 1) is a naturally occurring pentacyclic triterpenoid [1] which exhibits various biological activities, such as anti-HIV, anti-inflammatory, antioxidant, antiretroviral and antibacterial properties [2-6], Mostly recently, it has been employed as a potential anticancer agent by inhibition of topoisomerase [7-11]. BA, which is present as one of the major effective components of many traditional Chinese medicines [12], is widely distributed in the plant kingdom throughout the world [13]. BA has also been reported to inhibit growth of cancer cells, without affecting normal cells [14,15] and its lack of cytotoxic activity has been demonstrated in human astrocytes, human dermal fibroblasts, peripheral blood lymphoblasts and animal studies [16,17]. In 1995, BA was reported as a highly selective growth inhibitor [18] of human melanoma, neuroectodermal and malignant tumor cells and was reported to induce apoptosis in these cells [19]. At the same time, a large number of BA derivatives have been synthesized to improve anti-HIV activity [20,21], to reduce the organ toxic effect of antitumor drugs [22] and to be evaluated as new anticancer agents [23]. Researchers have demonstrated that the presence of a polar substituent at the C-3 position was essential for the pharmacological activities of pentacyclic triterpenes [24]. According to the structure–activity relationship, a hydrogen donor group at ether C-3 or C-28 position could improve cell proliferation inhibition significantly when an acetyl group was present at the C-3 position. Kvasnia et al found that the antitumor activities of BA derivatives where acyl groups were introduced at the C-3 position as well as C-28 positions were improved significantly [25]. In recent years, the drug in which amino alkyl groups were introduced has attracted considerable attention. The studies have shown that incorporation of amino alkyl groups has led to unexpected improvements in the anti-HIV or antitumor activities of compounds [26].

In order to search for BA derivatives with high antitumor bioactivity, we performed herein synthesis of new pentacyclic triterpene derivatives, particularly those with substituents at the 28-COOH position. The antiproliferative activities of the derivatives were evaluated against MGC-803, PC3, A375, Bcap-37 and A431 cell lines using MTT (thiazolyl blue tetrazolium bromide) assay *in vitro*. The results showed that some of the compounds displayed moderate to high levels of antitumor activities with most of new exhibiting more potent inhibitory activities compared to BA. The IC_50_ values of compound 3c with the five cancer cell lines were 2.3, 4.6, 3.3, 3.6, and 4.3 μM, respectively. Furthermore, experimental results of fluorescent staining and flow cytometry analysis (FCM) indicated that compound 3c could induce apoptosis in MGC-803 and PC3 cell lines to the extent 37.38% and 33.74% respectively after 36 h of treatment at 10 μM. These apoptosis ratios were more compared with the positive controls HCPT (Hydroxycamptothecin).

## Results and discussion

### Chemistry

**Scheme 1:**
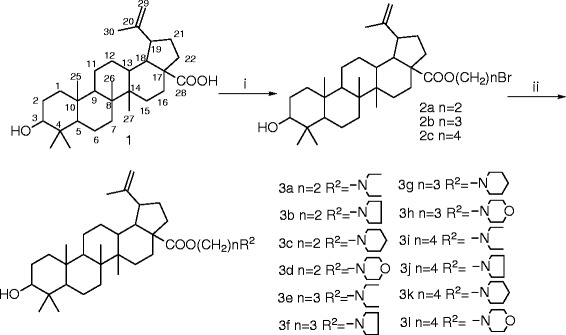
**Synthetic route to BA derivatives.** Reagents and conditions: (**i**) BrCH_2_CH_2_Br, BrCH_2_CH_2_CH_2_Br, or BrCH_2_(CH_2_)_2_CH_2_Br, K_2_CO_3_, DMF, r.t.; (**ii**) amine, K_2_CO_3_, DMF, r.t.

**Scheme 2:**
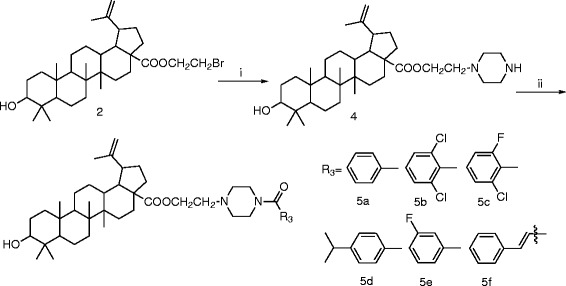
**Synthetic route to BA derivatives.** Reagents and conditions: (**i**) piperazine, K_2_CO_3_, DMF, 80 °C; (**ii**) EDCI, HOBT, R^3^COOH, DCM, r.t.

The synthesis of BA derivatives were summarized in Schemes 1- 2. The structure modification on BA used as the lead compound was done at the C-28 position. BA was acetylated with 1,2-dibromoethane, 1,3-dibromopropane, or 1,4-dibromobutane in the presence of K_2_CO_3_ in DMF at room temperature to give the compounds 2a-2c with high yield. Then, the compounds 2a-2c were reacted with corresponding amines to give the nitrogen-containing derivatives 3a-3l, respectively (Schemes 1). Compound 2a was treated with piperazine in DMF in the presence of K_2_CO_3_ at 80 ^o^C, and reacted with aromatic carboxylic acids to yield compounds 5a-5f (Schemes 2). All the compounds were fully characterized by their spectra data [see Additional file 1].

### Biological activity

The *in vitro* antitumor activities were evaluated for the synthesized compounds against the MGC-803, PC3, A375, Bcap-37, and A431 cell lines. BA, ADM (Adriamycin), HCPT, and the compounds were dissolved in DMSO. ADM and HCPT were used as positive controls, and the negative control cells were treated with culture medium containing 0.1% DMSO. When the IC_50_ (BA derivatives) could not reach the highest concentration, > 20 μM was employed for our study. Each experiment was repeated at least three times. The results were summarized in Table 1.

**Table 1:** Inhibitory activity of BA and derivatives on different cancer cells proliferation

**Compound**	**IC**_**50**_**(μM) **^**a **^**values against five human carcinoma cells**				
	**MGC-803**^**b**^	**PC3**^**c**^	**A375**^**d**^	**Bcap-37**^**e**^	**A431**^**f**^
1	41.2 ± 0.6	67.2 ± 0.5	> 100	> 100	15.7 ± 0.2
2a	> 20	> 20	> 20	> 20	> 20
2b	> 20	> 20	> 20	> 20	> 20
2c	> 20	> 20	> 20	> 20	> 20
3a	3.5 ± 0.5	5.6 ± 0.9	3.4 ± 0.1	5.5 ± 0.8	4.8 ± 0.2
3b	3.4 ± 0.2	4.2 ± 0.2	6.0 ± 0.4	7.6 ± 0.5	5.7 ± 0.2
3c	2.3 ± 0.2	4.6 ± 0.5	3.3 ± 0.4	3.6 ± 1.2	4.3 ± 0.3
3d	7.6 ± 2.1	8.6 ± 0.2	12.4 ± 1.1	10.5 ± 0.5	11.8 ± 0.6
3e	4.0 ± 0.9	3.8 ± 1.1	4.4 ± 0.5	3.0 ± 1.1	5.6 ± 0.1
3f	4.3 ± 0.2	2.8 ± 0.4	2.7 ± 0.3	5.2 ± 0.1	3.5 ± 0.6
3g	15.7 ± 0.5	13.4 ± 0.9	11.3 ± 0.6	16.2 ± 0.5	13.3 ± 0.4
3h	12.7 ± 1.2	15.0 ± 0.5	11.6 ± 0.5	12.5 ± 0.5	9.6 ± 0.9
3i	5.6 ± 0.4	7.9 ± 0.5	7.7 ± 0.3	6.8 ± 1.2	5.7 ± 0.2
3j	7.6 ± 0.6	13.4 ± 0.7	12.8 ± 0.4	7.5 ± 0.6	10.5 ± 1.5
3k	16.7 ± 0.5	17.1 ± 0.2	> 20	> 20	15.7 ± 0.7
3l	11.8 ± 0.3	12.7 ± 1.5	13.9 ± 0.8	9.43 ± 0.8	7.0 ± 0.4
5a	8.8 ± 0.8	10.1 ± 0.5	12.5 ± 0.6	9.4 ± 0.7	6.3 ± 0.8
5b	13.4 ± 0.1	14.5 ± 0.4	15.3 ± 2.3	16.7 ± 0.8	12.9 ± 1.2
5c	17.3 ± 0.4	> 20	> 20	> 20	> 20
5d	> 20	18.9 ± 0.3	> 20	> 20	16.3 ± 0.1
5e	> 20	> 20	> 20	> 20	> 20
5f	15.4 ± 0.6	> 20	> 20	> 20	> 20
HCPT	29.1 ± 2.6	34.5 ± 1.5	27.8 ± 1.2	28.1 ± 1.0	23.4 ± 0.7
ADM	0.7 ± 0.2	0.6 ± 0.1	1.0 ± 0.6	1.2 ± 0.2	1.1 ± 0.1

^a^ Agent concentration (micromolar) that inhibited cell growth by 50% 72 h after treatment; ^b^ Human gastric cancer; ^c^ Prostate cancer; ^d^ Malignant melanoma; ^e^ Breast cancer; ^f^ Epidermoid carcinoma.

As shown in Table 1, most of the modified compounds esterified at the 28-COOH with selected amino groups displayed significant further improvement of the cell growth inhibition. Compounds 3a-3f and 3i-3j presented strong inhibition against the above cancer cell lines. For example, the compound 3c showed considerable activity against the cancer cell lines (The IC_50_ values, 2.3, 4.6, 3.3, 3.6, and 4.3 μM, respectively). At the same time, it was found that active compounds showed antitumor activities against broader spectrum of tumors. Based on results we could summarize that the antiproliferative effect was significantly changed as the length of the carbon chain was increased (2 > 3 > 4), such as compounds 3a, 3e, and 3i. However, when the 28-COOH esterified with dibromoalkane, compounds 2a-2c had lower antitumor effects.

In addition, the compounds containing acyl piperazine moiety at C-28 did not display higher inhibitory activity than the other, and some compounds only had moderate antitumor activities.

It has been reported that BA and its derivatives induce apoptosis and growth inhibition in certain cancer cell lines [27,28]. In the present study, compound 3c was selected to analyze the mechanism of growth inhibition of MGC-803 and PC3 cell lines by following assays.

The morphologic changes in the cell after treatment with compound 3c were assessed by fluorescene microscopy after staining with AO/EB. AO permeates all cells and makes the nuclei appear green. EB is only taken up by cells when cytoplasmic membrane integrity is lost, and stains the nucleus with red and also dominates over AO. Thus live cells have a normal green nucleus; early apoptotic cells have bright green nucleus with condensed or fragmented chromatin; late apoptotic cells display condensed and fragmented orange chromatin; cells that died from direct necrosis have a structurally normal orange nucleus [29]. With HCPT and BA used as positive control at 10 μM for 48 h, the compound 3c at 5 μM on MGC-803 and PC3 cells from 12 to 48 h was detected *via* AO/EB staining.

**Figure 1:**
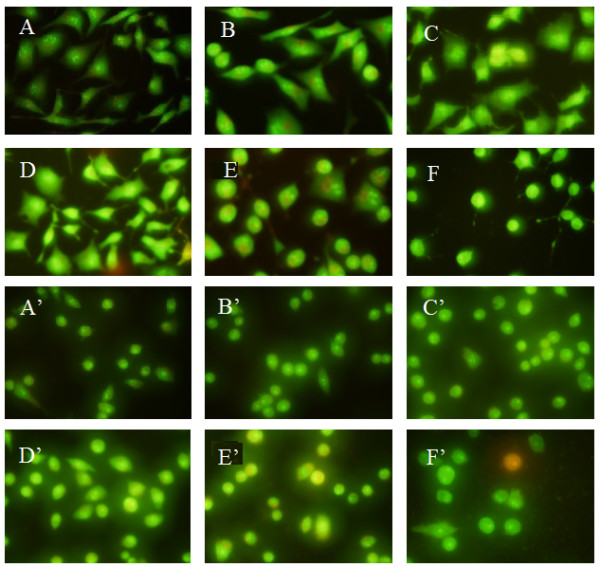
**The AO/EB staining of compound 3c in tumor cells.** For MGC-803 group, **A**: negative control; **B** and **C**: treated with HCPT and BA (10 μM each) as positive for 48 h; **D**, **E**, and **F**: treated with compound 3c (5 μM) for 12 h, 24 h, and 48 h, respectively. For PC3 cells group, A’: negative control; B’ and C’: treated with HCPT and BA (10 μM each) as positive for 48 h; D’, E’, and F’: treated with compound 3c (5 μM) for 12 h, 24 h, and 48 h, respectively.

As can be seen in Figure 1, green yellow or orange dots were detected in the HCPT after 48 h. Cells under BA treatment changed only minimally and pycnosis could only be seen after 48 h. And the cells treated with compound 3c from 12 to 48 h and BA for 24 h had changed. Yellow and orange dots in MGC-803 and PC3 cells showed early and late apoptotic cells, and the appearance of little red cells indicated that compound 3c was associated with low cytotoxicity. These findings show that compounds 3c could induce apoptosis with low cytotoxicity.

Hoechst 33258 is a hydrophilic and fluorescent probe only when bounded the DNA of cells, and live cells with uniformly light blue nuclei were treated with Hoechst 33258 [30]. The Hoechst 33258 staining showed apoptosis in all four types of cells, which were characterized by cytoplasmic and nuclear shrinkage, chromatin condensation and apoptosis body [31]. With HCPT and BA as positive control at 10 μM for 48 h, the compound **3c** at 5 μM on MGC-803 and PC3 cells from 12 to 48 h was detected *via* Hoechst 33258 staining.

**Figure 2:**
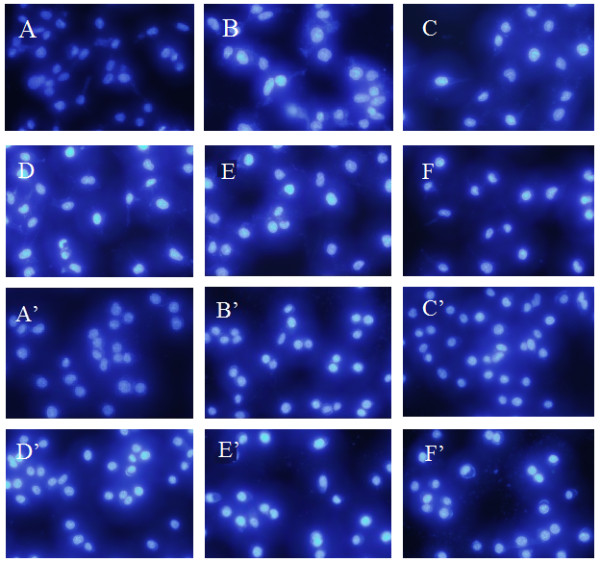
**The Hoechst 33258 staining of compound 3c in tumor cells.** For MGC-803 group, **A**: negative control; **B** and **C**: treated with HCPT and BA (10 μM each) as positive for 48 h; **D**, **E**, and **F**: treated with compound 3c (5 μM) for 12 h, 24 h, and 48 h, respectively. For PC3 cells group, A’: negative control; B’ and C’: treated with HCPT and BA (10 μM each) as positive for 48 h; D’, E’, and F’: treated with compound 3c (5 μM) for 12 h, 24 h, and 48 h, respectively.

As can be seen in Figure 2, the cells of the negative group (DMSO) were normal blue. However, the cells of HCPT group appeared compact condensed, and crescent-shaped. The cells exhibited strong blue fluorescence, revealing the typical apoptosis characteristics. The cells treated with compound 3c from 12 to 48 h and BA for 48 h had changed. The cell nuclei appeared to be highly condensed and crescent-shaped, indicating that compound **3c** induced apoptosis against MGC-803 and PC3 cell lines. These results were identical with the previous AO/EB double staining.

TUNEL is a common method for detecting DNA fragmentation that results from apoptotic signaling cascades. The assay relies on the presence of nicks in the DNA which can be identified by terminal deoxynucleotidyl transferase or TdT, an enzyme that can catalyze the addition of dUTPs that are secondarily labeled with a marker. It may also label cells that have suffered severe DNA damage. Under a fluorescence microscope, the cells were observed with brown precipitate were the result of positive apoptosis. With HCPT and BA as positive control at 10 μM for 48 h, the compound 3c at 5 μM against MGC-803 and PC3 cells from 12 to 48 h was detected *via* TUNEL assay.

**Figure 3:**
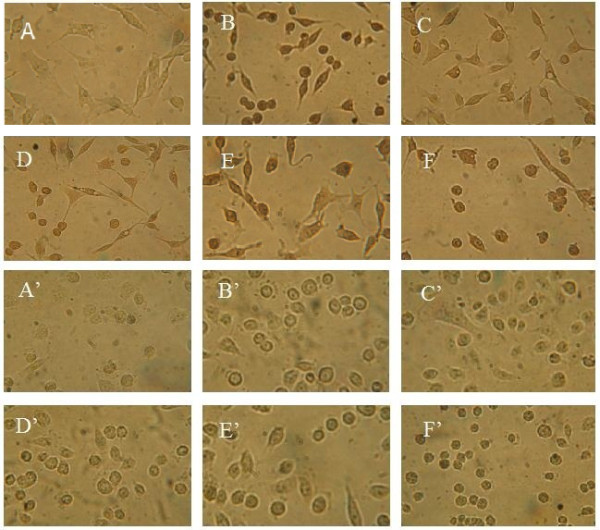
**The TUNEL assay of compound 3c in tumor cells.** For MGC-803 group, **A**: negative control; **B** and **C**: treated with HCPT and BA (10 μM each) as positive for 48 h; **D**, **E**, and **F**: treated with compound 3c (5 μM) for 12 h, 24 h, and 48 h, respectively. For PC3 cells group, A’: negative control; B’ and C’: treated with HCPT and BA (10 μM each) as positive for 48 h; D’, E’, and F’: treated with compound 3c (5 μM) for 12 h, 24 h, and 48 h, respectively.

As can be seen in Figure 3, the cells of the negative group (DMSO) did not appear as brown precipitates, whereas the other groups, namely, HCPT, appeared as brown precipitates. The cells treated with compound 3c from 12 to 48 h and BA for 48 h had changed. Therefore, it can be further concluded that compound 3c induced apoptosis against MGC-803 and PC3 cells. The results were identical with the previous experiment.

**Figure 4:**
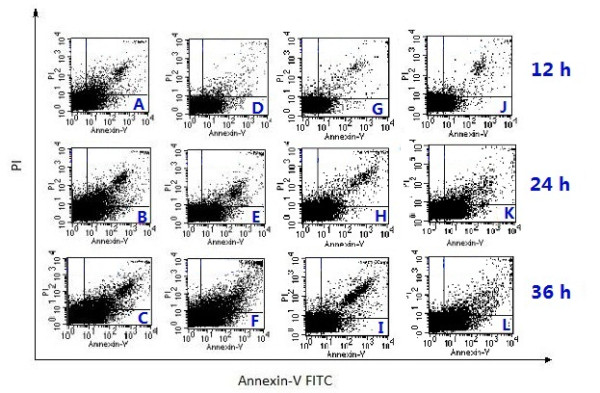
**Annexin V/PI apoptosis ratio detection assay.** The appearance of apoptosis cells was detected by flow cytometry using Annexin V/PI staining in the figure. (**A**, **B**, and **C**) MGC-803 cells were treated with HCPT (10 μM) from 12 to 36 h; (**D**, **E**, and **F**) MGC-803 cells were treated with compound 3c (10 μM) from 12 to 36 h; (**G**, **H**, and **I**) PC3 cells were treated with HCPT (10 μM) from 12 to 36 h; (**J**, **K**, and **L**) PC3 cells were treated with compound 3c (10 μM) from 12 to 36 h.

**Figure 5:**
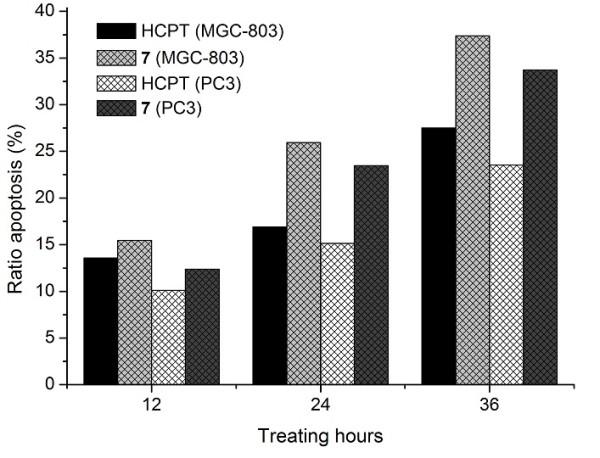
**The apoptosis ratios of MGC-803 and PC3 cells treated with compound 3c assessed by FCM.** These cells were treated with HCPT (10 μM) and compound 3c (10 μM) for 12, 24, and 36 h. The apoptosis ratio includes the early and late apoptosis ratios.

The apoptosis ratios induced by compound 3c in tumor cells were quantitatively assessed by FCM. In early apoptotic cells, phosphatidylserine (PS) which distributed inside the lipid bilayer in the normal cells was transferred from the inside of the cell membrane to the outside. Annexin V was a calcium-dependent phospholipid binding protein that has a high affinity for the phophatidylserine PS, a plasma membrane phospholipid, used to detect early apoptotic cells. PI (Propidine Iodide) is an intercalating agent and a fluorescent molecule with a molecular mass of 668.4 Da that can be used to stain cells that had lost membrane integrity. So, the different periods of apoptotic cells could be distinguished when Annexin V matched with PI: necrotic cells (the upper left quadrant, Annexin^-^/PI^+^), late apoptotic cells (the upper right quadrant, Annexin^+^/PI^+^), intact cells (the lower left quadrant, Annexin^-^/PI^-^) and early apoptotic cells (the lower right quadrant, Annexin^+^/PI^-^) [32]. As shown in Figure 4, with HCPT as positive control, compound 3c (10 μM) could induce apoptosis of MGC-803 and PC3 cells, and highest apoptosis ratios, 37.38% and 33.74% for compound 3c, were obtained after 36 h of treatment at a concentration of 10 μM. Furthermore, as shown in Figure 5 the apoptosis of MGC-803 and PC3 cells which were treated with compound 3c increased gradually in a time-dependent manner.

## Conclusions

BA is an important natural product, and more and more studies have shown that BA has significant anticancer activity against various kinds of cell lines *in vitro*. In our study, a series of BA derivatives were designed, synthesized and evaluated their antitumor activities against the MGC-803, PC3, A375, Bcap-37, and A431 cell lines. Our data suggest that the C-28 amino substituted BA derivatives possess stronger antiproliferative ability. Eight compounds (3a-3f, 3i-3j) showed significant cell growth inhibition activity against the five cancer cell lines. The IC_50_ values of compound 3c against the five cancer cell lines were 2.3, 4.6, 3.3, 3.6, and 4.3 μM, respectively. However, the compounds containing an acyl piperazine moiety at C-28 did not display higher inhibitory activity, and some compounds only had moderate antitumor activity, but still more active than the parent BA.

In addition, the apoptosis-inducing activity of compound 3c in MGC-803 and PC3 cell lines was investigated by following assays: AO/EB staining, Hoechst 33258 staining, TUNEL assay and FCM. The preliminary mechanistic studies indicated that the compound 3c may inhibit cell growth by inducing apoptosis. The apoptosis ratio reached 37.38% and 33.74% after 36 h of treatment at 10 μM, higher than the ratios observed for the positive control HCPT (27.49% and 23.53% respectively). These findings provide a very powerful incentive for further research on the chemical modification and structure-activity relationships of BA and other triterpenoid acids.

## Experimental

### General procedures and reagents

BA with more than 98% purity was purchased from Zhejiang Tiancao Biotech Co., Ltd. Reagents of analytical grade were obtained from Yuda Chemistry Co., Ltd., and used without further purification unless otherwise noted. Infrared spectra were recorded on a Bruker VECTOR22 spectrometer in KBr disks. ^1^H-NMR and ^13^C-NMR were recorded using a JEOL-ECX500 spectrometer at 22°C, with tetramethylsilane as the internal standard and CDCl_3_ as the solvent. Column chromatography was performed using silica gel (200–300 meshes) (Qingdao Marine Chemistry Co., Qingdao, China)

### Cell lines and culture

MGC-803, PC3, A375, Bcap-37 and A431 cell lines were obtained from the Institute of Biochemistry and Cell Biology, China Academy of Science. MGC-803 is gastric cancer cell line, PC3 is prostate cancer cell line, A375 is malignant melanoma cell line, Bcap-37 is breast cancer cell line, and A431 is epidermoid carcinoma cell line. The entire cancer cell lines were maintained in the RPMI 1640 medium. They were supplemented with 10% heat-inactivated fetal bovine serum (FBS) in a humidified atmosphere of 5% CO_2_ at 37°C. All cell lines were maintained at 37°C in a humidified 5% carbon dioxide and 95% air incubator.

### MTT assays

All tested compounds were dissolved in DMSO and subsequently diluted in the culture medium before treatment of the cultured cells. When the cells were 80-90% confluent, they were harvested by treatment with a solution containing 0.25% trypsin, thoroughly washed and resuspended in supplemented growth medium. Cells (2×10^3^/well) were plated in 100 μL of medium/well in 96-well plate. After incubations overnight, the cells were treated with different concentrations of extracts in RPMI 1640 with 10% FBS for 72 h. In parallel, the cells treated with 0.1% DMSO served as negative control and ADM (Adriamycin) as positive control. Finally, 100 μL of MTT was added, and the cells were incubated for 4 h. The MTT-formazan formed by metabolically viable cells was dissolved in 100 μL of SDS for 12 h. The absorbance was then measured at 595 nm with a microplate reader, which is directly proportional to the number of living cells in culture.

### AO/EB staining

When the cells were 80-90% confluent, they were harvested by treatment with a solution containing 0.25% trypsin, thoroughly washed and resuspended in supplemented growth medium. The cells were seeded at a concentration of 5 × 10^4^ cell/mL in a volume of 0.8 mL on a sterile cover slip in 6-well tissue culture plates. Following incubation, the medium was removed and replaced with fresh medium plus 10% FBS and then supplemented with compounds. After the treatment period, the cover slip with monolayer cells was inverted on the glass slide with 20 μL of AO/EB stain (100 μg/mL). The fluorescence was read using fluorescence microscope.

### Hoechst 33258 staining

When the cells were 80-90% confluent, they were harvested by treatment with a solution containing 0.25% trypsin, thoroughly washed and resuspended in supplemented growth medium. The cells were seeded at a concentration of 5 × 10^4^ cell/mL in a volume of 0.8 mL on a sterile cover slip in 6-well tissue culture plates, and were treated with compounds for a certain range of treatment time. The culture medium containing compounds was removed, and the cells were fixed in 4% paraformaldehyde for 10 min. The cells were washed twice with PBS, and were consequently stained with 0.5 mL of Hoechst 33258 staining for 5 min. The stained nuclei were washed twice with PBS, and were consequently observed under fluorescence microscope at 350 nm excitation and 460 nm emissions.

### TUNEL assay

When the cells were 80-90% confluent, they were harvested by treatment with a solution containing 0.25% trypsin, thoroughly washed and resuspended in supplemented growth medium. The cells were seeded at a concentration of 5 × 10^4^ cell/mL in a volume of 0.8 mL on a sterile cover slip in 6-well tissue culture plates, and were treated with compounds for a certain range of treatment time. A) The MGC-803 and PC3 cells grown in 6-well tissue culture plates were washed with PBS and fixed in 4% paraformaldehyde for 40 min. B) The cells were washed once with PBS, and were then permeabilized with immunol staining wash buffer for 2 min on ice. The cells were rewashed once with PBS, and subsequently incubated in 0.3% H_2_O_2_ in methanol at room temperature for 20 min to inactivate the endogenous peroxidases, after which the cells were washed thrice with PBS. C) Thereafter, the cells were incubated with 2 μL of TdT-enzyme and 48 μL of Biotin-dUTP per specimen for 60 min at 37°C. The cells were terminated for 10 min, and were subsequently incubated with streptavidin-HRP (50 μL per specimen) conjugate diluted at 1:50 in sample diluent for 30 min. D) The cells were washed three times with PBS, and were then incubated with diaminobenzidine solution (200 μL per specimen) for 10 min. E) The cells were rewashed twice with PBS, and were finally imaged under biological microscope.

### Flow cytometry analysis

Prepared MGC-803 and PC3 cells (1×10^6^/mL) were washed twice with cold PBS and then re-suspended gently in 500 μL binding buffer. Then the cells were stained in 5 μL Annexin V-FITC and shaked well. 5 μL PI was added to these cells and incubated for 20 min in a dark place, analyzed by FACS.

### Statistical analysis

All statistical analyses were performed using SPSS 10.0, and the data were analyzed using one-way ANOVA. The mean separations were performed using the least significant difference method. Each experiment was performed in triplicate, and all experiments were run thrice and yielded similar results. Measurements from all the replicates were combined, and the treatment effects were analyzed.

## Competing interests

We have no competing interesting.

## Author’s contributions

SY synthesized the compounds and carried out most of the bioassay experiments. NL and HL did part of the bioassay experiments. WX took part in the compound structural elucidation and bioassay experiments. DH and LJ carried out some structure elucidation experiments. QZ assisted in structural elucidation experiments. Prof. SY is the co-corresponding author for this work. All authors read and approved the final manuscript.

## Supplementary Material

Additional file 1 **Experimental details and data of BA derivatives.** Which includes the experimental procedure, spectroscopic data, and copies of ^1^H NMR and ^13^C NMR of selected compounds. Click here for file
