# Supplementary material for: Design, synthesis and biological evaluation of novel betulinic acid derivatives
Source: Chem Cent J. 2012 Nov 23;6:141. doi: 10.1186/1752-153X-6-141 (PMC3541990; doi:10.1186/1752-153X-6-141)

Additional file 1- **Supporting Information**

# Design, synthesis and antitumor activity evaluation of novel betulinic acid derivatives

Shengjie Yang, Na Liang, Hu Li, Wei Xue, Deyu Hu, Linhong Jin, Qi Zhao, and Song Yang*

State Key Laboratory Breeding Base of Green Pesticide and Agricultural Bioengineering, Key Laboratory of Green Pesticide and Agricultural Bioengineering, Ministry of Education, Guizhou University, Guiyang 550025, P.R. China

*Corresponding authors. Ctr for R&D of Fine Chemicals, Guizhou University, Huaxi St., Guiyang, China 550025. Tel.: +86 851 829 2171; fax: +86 851 829 2170.

E-mail addresses: yangstg2003@gmail.com

Email addresses:

Shengjie Yang: yangsj501904006@126.com

Na Liang: liangna8703@163.com

Hu Li: lhpesticide@163.com

Wei Xue: shouldww@126.com

Deyu Hu: fcc.dyhu@gzu.edu.cn

Linhong Jin: fcc.lhjin@gzu.edu.cn

Qi Zhao: 406587521@qq.com

Song Yang*: yangstg2003@gmail.com

## Synthesis

***General procedure for compounds 2-4.***

BA (1 mmol) and K_2_CO_3_ (2 mmol) were added to DMF (25 mL) and stirred at room temperature for 10 min, after which 1,2-dibromoethane, 1,3-dibromopropane, or 1,4-dibromobutane (4 mmol) was added. After being stirred for another 24 h, the reaction mixture was poured onto 100 mL of distilled water and partitioned with DCM (3×25 mL). The organic layer was washed with saturated sodium chloride, dried over Na_2_SO_4_ and purified via silica gel column chromatography with petroleum ether/ethyl acetate to obtain compound **2a-2c**.

Compound **2a:** Yield: 72.9%; white powder, mp: 145-147 ^o^C; IR (KBr, cm^-1^): ν_max_ 3445, 2923, 1678, 1322, 1095, 882. ^1^H NMR (CDCl_3_, 500 MHz) δ: 4.72 (1H, brs, Hb-29), 4.59 (1H, br s, Ha-29), 4.39 (2H, *J* = 11.5 Hz, CH_2_), 3.52 (2H, *J* = 5.5 Hz, CH_2_), 3.17 (1H, dd, *J* = 4.5Hz, H-3a), 3.02 (1H, m, H-19), 1.70 (3H, s, H-30), 1.03 (3H, s, H-27), 0.97 (6H, s, H-23, H-26), 0.89 (3H, s, H-25), 0.74 (3H, s, H-24); ^13^C NMR (CDCl_3_, 125 MHz) δ: 175.8 (C-28), 150.5 (C-20), 109.7 (C-29), 79.1 (C-3), 63.4 (CH_2_), 56.7 (C-17), 55.4 (C-5), 50.6 (C-9), 49.5 (C-18), 47.0 (C-19), 42.5 (C-14), 40.8 (C-8), 38.9 (C-4), 38.8 (C-1), 38.4 (C-13), 37.3 (C-22), 37.1 (C-10), 34.4 (C-7), 32.1 (C-16), 30.6 (C-21), 29.7 (C-15), 29.2 (CH_2_), 28.0 (C-23), 27.5 (C-2), 25.6 (C-21), 20.9 (C-11), 19.4 (C-30), 18.3 (C-6), 16.2 (C-26), 16.0 (C-25), 15.4 (C-24), 14.8 (C-27).

Compound **2b**: Yield: 76.2%; colorless oil; IR (KBr, cm^-1^): ν_max_ 3452, 2911, 1688, 1638, 1343, 1102, 884. ^1^H NMR (CDCl_3_, 500 MHz) δ: 4.71 (1H, br s, Hb-29), 4.59 (1H, br s, Ha-29), 4.21 (2H, m, CH_2_), 3.47 (2H, m, CH_2_), 3.17 (1H, dd, *J* = 5, 7.5Hz, H-3a), 3.00 (1H, m, H-19), 1.67 (3H, s, H-30), 0.97 (3H, s, H-27), 0.95 (6H, s, H-23, H-26), 0.90 (3H, s, H-25), 0.74 (3H, s, H-24); ^13^C NMR (CDCl_3_, 125 MHz) δ: 175.9 (C-28), 150.5 (C-20), 109.8 (C-29), 79.0 (C-3), 61.6 (CH_2_), 56.7 (C-17), 55.4 (C-5), 50.6 (C-9), 49.5 (C-18), 47.1 (C-19), 42.5 (C-14), 40.8 (C-8), 38.9 (C-4), 38.8 (C-1), 38.4 (C-13), 37.3 (C-22), 37.1 (C-10), 34.4 (C-7), 32.2 (C-16), 31.7 (CH_2_), 30.7 (C-21), 29.7 (C-15), 29.6(CH_2_), 28.0(C-23), 27.5(C-2), 25.6 (C-21), 20.9 (C-11), 19.4 (C-30), 18.3 (C-6), 16.2 (C-26), 16.1 (C-25), 15.5 (C-24), 14.8 (C-27).

Compound **2c**: Yield: 80.3%; colorless oil; IR (KBr, cm^-1^): ν_max_ 3466, 2952, 1695, 1642, 1373, 1021, 885. ^1^H NMR (CDCl_3_, 500 MHz) δ: 4.71 (1H, br s, Hb-29), 4.59 (1H, br s, Ha-29), 4.10 (2H, m, CH_2_), 3.46 (2H, m, CH_2_), 3.17 (1H, dd, *J* = 5.5, 7.5Hz, H-3a), 2.97 (1H, m, H-19), 1.81 (2H, m, CH_2_), 1.66 (3H, s, H-30), 0.99 (3H, s, H-27), 0.94 (6H, s, H-23, H-26), 0.89 (3H, s, H-25), 0.74 (3H, s, H-24); ^13^C NMR (CDCl_3_, 125 MHz) δ: 176.1 (C-28), 150.7 (C-20), 109.6 (C-29), 79.2 (C-3), 62.8 (CH_2_), 56.7 (C-17), 55.4 (C-5), 50.5 (C-9), 48.9 (C-18), 47.2 (C-19), 42.5 (C-14), 40.8 (C-8), 38.9 (C-4), 38.6 (C-1), 38.4 (C-13), 37.2 (C-22), 37.6 (C-10), 34.4 (C-7), 33.6 (CH_2_), 32.8 (C-16), 30.7 (C-21), 29.7 (C-15), 29.5 (CH_2_), 28.4 (C-23), 27.5 (C-2), 27.0 (CH_2_), 25.6 (C-21), 20.9 (C-11), 19.7 (C-30), 18.3 (C-6), 16.9 (C-26), 16.0 (C-25), 15.5 (C-24), 14.1 (C-27).

***General procedure for compounds 3a-3l.***

The compounds **2a**, **2b** or **2c** (1 mmol) and K_2_CO_3_ (2 mmol) were assed to DMF (10 mL) and stirred at room temperature for 10 min, after saturated nitrogen heterocyclic rings (2 mmol) was added. After being stirred for 24 h, the reaction mixture was poured into the 100 mL distilled water and partitioned with DCM (3×30 mL). The organic layer was washed with saturated sodium chloride, dried over Na_2_SO_4_, and purified via silica gel column chromatography with CHCl_3_/MeOH (10:1, v/v) to obtain compounds **3a-3l**.

Compound **3a**: Yield: 75.5%; yellow colorless oil; IR (KBr, cm^-1^): ν_max_ 3433, 2946, 2865,1683, 1638, 884. ^1^H NMR (CDCl_3_, 500 MHz) δ: 4.70 (1H, br s, Hb-29), 4.57 (1H, br s, Ha-29), 4.15 (2H, m, CH_2_), 3.17 (1H, dd, *J* = 4, 7.5 Hz, H-3a), 2.99 (1H, m, H-19), 2.72 (2H, t, *J* = 6 Hz, CH_2_), 2.60 (4H, m, CH_2_), 1.68 (3H, s, H-30), 1.02(6H, t, *J* = 12.5 Hz, CH_3_), 0.96 (6H, s, H-27), 0.88 (3H, s, H-23, H-26), 0.78 (3H, s, H-25), 0.73 (3H, s, H-24); ^13^C-NMR (CDCl_3_, 125 MHz) δ: 176.1 (C-28), 150.7 (C-20), 109.6 (C-29), 79.0 (C-3), 61.7 (CH_2_), 56.6 (C-17), 55.4 (C-5), 51.0(CH_2_), 50.6 (C-9), 49.4 (C-18), 47.4(CH_2_), 46.9 (C-19), 42.5 (C-14), 40.8 (C-8), 38.9 (C-4), 38.8 (C-1), 38.2 (C-13), 37.2 (C-22), 36.9 (C-10), 34.4 (C-7), 32.2 (C-16), 30.6 (C-21), 29.7 (C-15), 28.1 (C-23), 27.5 (C-2), 25.6 (C-21), 20.9 (C-11), 19.4 (C-30), 18.4 (C-6), 16.2 (C-26), 16.1 (C-25), 15.5 (C-24), 14.8 (C-27), 11.7 (CH_3_).

Compound **3b**: Yield: 76.8%; yellow colorless oil; IR (KBr, cm^-1^): ν_max_ 3441, 2950, 1688, 1634, 1382, 885. ^1^H NMR (CDCl_3_, 500 MHz) δ: 4.73 (1H, br s, Hb-29), 4.60 (1H, br s, Ha-29), 4.12 (2H, m, CH_2_), 3.18 (1H, dd, *J* = 4.5, 7.5 Hz, H-3a), 3.00 (1H, m, H-19), 2.83 (1H, t, *J* = 6.5 Hz, CH_2_), 2.70 (4H, br s, CH_2_), 1.72 (4H, m, CH_2_), 1.68 (3H, s, H-30), 0.96 (6H, s, H-27), 0.91 (3H, s, H-23, H-26), 0.82 (3H, s, H-25), 0.76 (3H, s, H-24); ^13^C NMR (CDCl_3_, 125 MHz) δ: 175.7 (C-28), 150.5 (C-20), 109.7 (C-29), 77.4 (C-3), 60.2 (CH_2_), 56.6 (C-17), 56.5 (CH_2_), 55.4 (C-5), 54.0 (CH_2_), 50.6 (C-9), 49.4 (C-18), 46.9 (C-19), 42.5 (C-14), 40.8 (C-8), 38.9 (C-4), 38.8 (C-1), 38.2 (C-13), 37.2 (C-22), 36.9 (C-10), 34.4 (C-7), 32.2 (C-16), 30.6 (C-21), 29.7 (C-15), 28.1 (C-23), 27.5 (C-2), 25.6 (C-21), 24.6 (CH_2_), 20.9 (C-11), 19.4 (C-30), 18.4 (C-6), 16.2 (C-26), 16.1 (C-25), 15.5 (C-24), 14.8 (C-27).

Compound **3c**: Yield: 70.1%; yellow colorless oil; IR (KBr, cm^-1^): ν_max_ 3444, 2938, 2861, 1687, 1640, 880. ^1^H NMR (CDCl_3_, 500 MHz) δ: 4.70 (1H, br s, Hb-29), 4.56 (1H, br s, Ha-29), 4.12 (2H, m, CH_2_), 3.15 (1H, dd, *J* = 5, 10 Hz, H-3a), 2.98 (1H, m, H-19), 2.53 (2H, t, *J* = 4.5 Hz, CH_2_), 2.43 (4H, br s, CH_2_), 1.67 (3H, s, H-30), 1.54 (m, CH_2_), 0.99 (6H, s, H-27), 0.87 (3H, s, H-23, H-26), 0.77 (3H, s, H-25), 0.73 (3H, s, H-24); ^13^C NMR (CDCl_3_, 125 MHz) δ: 176.3 (C-28), 150.6 (C-20), 109.8 (C-29), 77.8 (C-3), 60.3 (CH_2_), 56.7 (CH_2_), 56.6 (C-17), 55.4 (C-5), 54.5 (CH_2_), 50.6 (C-9), 49.8 (C-18), 46.9 (C-19), 42.75 (C-14), 40.6 (C-8), 38.9 (C-4), 38.8 (C-1), 38.2 (C-13), 37.2 (C-22), 36.9 (C-10), 34.4 (C-7), 32.2 (C-16), 30.6 (C-21), 29.7 (C-15), 29.2 (CH_2_), 28.1 (C-23), 27.2 (C-2), 25.7 (C-21), 24.6 (CH_2_), 20.9 (C-11), 19.4 (C-30), 18.4 (C-6), 16.2 (C-26), 16.1 (C-25), 15.5 (C-24), 14.8 (C-27).

Compound **3d**: Yield: 79.8%; yellow colorless oil; IR (KBr, cm^-1^): ν_max_ 3450, 2927, 2863, 1681, 1642, 886. ^1^H NMR (CDCl_3_, 500 MHz) δ: 4.71(1H, br s, Hb-29), 4.59 (1H, br s, Ha-29), 4.20 (2H, m, CH_2_), 3.69 (4H, t, *J* = 6 Hz, CH_2_), 3.18(1H, dd, *J* = 6, 10.5 Hz, H-3a), 2.99 (1H, m, H-19), 2.61 (4H, t, *J* = 7 Hz, CH_2_), 2.50 (2H, br s, CH_2_), 1.68 (3H, s, H-30), 0.96 (6H, s, H-27), 0.90 (3H, s, H-23, H-26), 0.79 (3H, s, H-25), 0.74 (3H, s, H-24); ^13^C-NMR (CDCl_3_, 125 MHz) δ: 176.0 (C-28), 150.7 (C-20), 109.7 (C-29), 77.3 (C-3), 67.0 (CH_2_), 60.8 (CH_2_), 57.3 (CH_2_), 56.6 (C-17), 55.4 (C-5), 53.8 (CH_2_), 50.6 (C-9), 49.4 (C-18), 47.0 (C-19), 42.5 (C-14), 40.8 (C-8), 38.9 (C-4), 38.8 (C-1), 38.3 (C-13), 37.2 (C-22), 37.1 (C-10), 34.4 (C-7), 32.2 (C-16), 30.6 (C-21), 29.7 (C-15), 28.0 (C-23), 27.5 (C-2), 25.6 (C-21), 20.9 (C-11), 19.4 (C-30), 18.3 (C-6), 16.2 (C-26), 16.1 (C-25), 15.5 (C-24), 14.8 (C-27).

Compound **3e**: Yield: 77.3%; yellow colorless oil; IR (KBr, cm^-1^): ν_max_ 3457, 2949, 1682, 1646, 1364, 1053, 885. ^1^H NMR (CDCl_3_, 500 MHz) δ: 4.69 (1H, br s, Hb-29), 4.57 (1H, br s, Ha-29), 4.07 (2H, m, CH_2_), 3.12 (1H, dd, *J* = 5, 11.5 Hz, H-3a), 2.98 (1H, m, H-19), 2.50 (4H, m, CH_2_), 2.21 (2H, m, CH_2_), 1.68 (3H, s, H-30), 1.01 (6H, t, *J* = 13 Hz, CH_3_), 0.96 (6H, s, H-27), 0.89 (3H, s, H-23, H-26), 0.79 (3H, s, H-25), 0.73 (3H, s, H-24); ^13^C-NMR (CDCl_3_, 125 MHz) δ: 176.3 (C-28), 150.6 (C-20), 109.7 (C-29), 78.9 (C-3), 62.6 (CH_2_), 56.6 (CH_2_), 56.6 (C-17), 55.4 (C-5), 50.6 (C-9), 49.6 (C-18), 49.4 (CH_2_), 47.1 (C-19), 42.5 (C-14), 40.8 (C-8), 38.9 (C-4), 38.8 (C-1), 38.3 (C-13), 37.2 (C-22), 37.1 (C-10), 34.4 (C-7), 32.3 (C-16), 30.7 (C-21), 29.7 (C-15), 28.0 (C-23), 27.5 (C-2), 26.5 (CH_2_), 25.6 (C-21), 20.9 (C-11), 19.4 (C-30), 18.3 (C-6), 16.2 (C-26), 16.1 (C-25), 15.5 (C-24), 14.8 (C-27), 11.8 (CH_3_).

Compound **3f**: Yield: 82.1%; yellow colorless oil; IR (KBr, cm^-1^): ν_max_ 3451, 2967, 1687, 1633, 1386, 1038, 889. ^1^H NMR (CDCl_3_, 500 MHz) δ: 4.70 (1H, br s, Hb-29), 4.57 (1H, br s, Ha-29), 4.10 (2H, m, CH_2_), 3.15 (1H, dd, *J* = 5.5, 11 Hz, H-3a), 2.98 (1H, m, H-19), 2.48 (4H, m,CH_2_), 1.77 (m, CH_2_), 1.65 (3H, s, H-30), 1.36 (m, CH_2_), 0.99 (6H, s, H-27), 0.88 (3H, s, H-23, H-26), 0.78 (3H, s, H-25), 0.73 (3H, s, H-24); ^13^C NMR (CDCl_3_, 125 MHz) δ: 176.2 (C-28), 150.7 (C-20), 109.7 (C-29), 78.9 (C-3), 62.5 (CH_2_), 56.6 (C-17) 55.4 (C-5), 54.3 (CH_2_), 53.3 (CH_2_), 50.6 (C-9), 49.4 (C-18), 47.1 (C-19), 42.5 (C-14), 40.8 (C-8), 38.9 (C-4), 38.8 (C-1), 38.4 (C-13), 37.3 (C-22), 37.2 (C-10), 34.4 (C-7), 32.2 (C-16), 30.7 (CH_2_), 29.7 (C-21), 28.4 (C-15), 28.1 (C-23), 27.5 (C-2), 25.6 (C-21), 23.5 (CH_2_), 20.9 (C-11), 19.5 (C-30), 18.4 (C-6), 16.2 (C-26), 16.1 (C-25), 15.5 (C-24), 14.7 (C-27).

Compound **3g**: Yield: 74.6%; yellow colorless oil; IR (KBr, cm^-1^): ν_max_ 3512, 2944, 1702, 1397, 1051, 882. ^1^H NMR (CDCl_3_, 500 MHz) δ: 4.69 (1H, brs, Hb-29), 4.52 (1H, brs, Ha-29), 4.06 (2H, m, CH_2_), 3.15 (1H, dd, *J* = 4.5, 8.0 Hz, H-3a), 2.96 (1H, m, H-19), 2.35 (4H, m, CH_2_), 1.82 (m, CH_2_), 1.65 (3H, s, H-30), 1.55 (m, CH_2_), 0.99 (6H, s, H-27), 0.87 (3H, s, H-23, H-26), 0.77 (3H, s, H-25), 0.72 (3H, s, H-24); ^13^C NMR (CDCl_3_, 125 MHz) δ: 176.2 (C-28), 150.7 (C-20), 109.7 (C-29), 77.4 (C-3), 62.6 (CH_2_), 56.6 (C-17) 55.4 (C-5), 54.7 (CH_2_), 50.6 (C-9), 49.4 (C-18), 47.9 (CH_2_), 47.1 (C-19), 42.5 (C-14), 40.8 (C-8), 38.9 (C-4), 38.8 (C-1), 38.4 (C-13), 37.3 (C-22), 37.1 (C-10), 34.4 (C-7), 32.2 (C-16), 30.7 (CH_2_), 30.0 (C-21), 29.7 (C-15), 28.0 (C-23), 27.5 (C-2), 26.0 (CH_2_), 25.6 (C-21),24.5 (CH_2_), 20.9 (C-11), 19.5 (C-30), 18.4 (C-6), 16.2 (C-26), 16.1 (C-25), 15.5 (C-24), 14.7 (C-27).

Compound **3h**: Yield: 69.3%; yellow colorless oil; IR (KBr, cm^-1^): ν_max_ 3509, 2946, 1684, 1379, 1044, 882. ^1^H NMR (CDCl_3_, 500 MHz) δ: 4.74 (1H, br s, Hb-29), 4.57 (1H, br s, Ha-29), 4.14 (2H, m, CH_2_), 3.68 (4H, t, *J* = 10 Hz, CH_2_), 3.15 (1H, dd, *J* = 5, 8.5 Hz, H-3a), 2.98 (1H, m, H-19), 2.41 (6H, m, CH_2_), 1.65 (3H, s, H-30), 0.99 (6H, s, H-27), 0.89 (3H, s, H-23, H-26), 0.79 (3H, s, H-25), 0.72 (3H, s, H-24); ^13^C NMR (CDCl_3_, 125 MHz) δ: 176.2 (C-28), 150.6 (C-20), 109.7 (C-29), 78.9 (C-3), 67.0 (CH_2_), 62.2 (CH_2_), 56.6 (C-17) 55.7 (CH_2_), 55.4 (C-5), 53.7 (CH_2_), 50.6 (C-9), 49.4 (C-18), 47.1 (C-19), 42.5 (C-14), 40.8 (C-8), 38.9 (C-4), 38.7 (C-1), 38.3 (C-13), 37.2 (C-22), 37.1 (C-10), 34.4 (C-7), 32.2 (C-16), 30.7 (C-21), 29.7 (C-15), 28.1 (C-23), 27.5 (C-2), 25.9 (C-21), 25.5 (CH_2_), 20.9 (C-11), 19.5 (C-30), 18.4 (C-6), 16.2 (C-26), 16.1 (C-25), 15.5 (C-24), 14.7 (C-27).

Compound **3i**: Yield: 78.4 %; yellow colorless oil; IR (KBr, cm^-1^): ν_max_ 3448, 2934, 2855, 1683, 1716, 1345, 878. ^1^H NMR (CDCl_3_, 500 MHz) δ: 4.73(1H, br s, Hb-29), 4.59 (1H, br s, Ha-29), 4.11 (2H, m, CH_2_), 3.17 (1H, dd, *J* = 6.5, 11.5 Hz, H-3a), 3.01 (1H, m, H-19), 2.52 (4H, m, CH_2_), 2.26 (2H, m, CH_2_), 1.69 (3H, s, H-30), 1.37 (2H, m, CH_2_), 1.01 (6H, t, *J* = 13 Hz, CH_3_), 0.99 (6H, s, H-27), 0.91 (3H, s, H-23, H-26), 0.82 (3H, s, H-25), 0.76 (3H, s, H-24); ^13^C NMR (CDCl_3_, 125 MHz) δ: 176.2 (C-28), 150.6 (C-20), 109.7 (C-29), 77.4 (C-3), 63.9 (CH_2_), 56.6 (C-17), 55.4 (C-5), 52.4 (CH_2_), 50.6 (C-9), 49.4 (C-18), 47.1 (CH_2_), 46.9 (C-19), 42.5 (C-14), 40.8 (C-8), 38.9 (C-4), 38.8 (C-1), 38.3 (C-13), 37.2 (C-22), 37.1 (C-10), 34.4 (C-7), 32.3 (C-16), 30.7 (C-21), 29.7 (C-15), 28.1 (C-23), 27.5 (C-2), 26.9 (CH_2_), 25.6 (C-21), 23.5 (CH_2_), 20.9 (C-11), 19.4 (C-30), 18.3 (C-6), 16.2 (C-26), 16.1 (C-25), 15.5 (C-24), 14.8 (C-27), 11.8 (CH_3_).

Compound **3j**: Yield: 75.6%; yellow colorless oil; IR (KBr, cm^-1^): ν_max_ 3422, 2930, 2871, 1678, 1642, 1078, 881. ^1^H NMR (CDCl_3_, 500 MHz) δ: 4.70 (1H, br s, Hb-29), 4.59 (1H, br s, Ha-29), 4.11 (2H, m, CH_2_), 3.19 (1H, dd, *J* = 4, 8.5 Hz, H-3a), 3.09 (1H, m, H-19), 2.45 (6H, m, CH_2_), 1.84 (2H, m, CH_2_), 1.67 (3H, s, H-30), 1.58 (2H, m, CH_2_), 1.35 (2H, m, CH_2_), 0.96 (6H, s, H-27), 0.87 (3H, s, H-23, H-26), 0.78 (3H, s, H-25), 0.71 (3H, s, H-24); ^13^C NMR (CDCl_3_, 125 MHz) δ: 176.2 (C-28), 150.7 (C-20), 109.7 (C-29), 78.9 (C-3), 63.9 (CH_2_), 56.6 (C-17), 56.2(CH_2_), 55.4 (C-5), 54.3 (CH_2_), 50.6 (C-9), 49.4 (C-18), 47.1 (C-19), 42.5 (C-14), 40.8 (C-8), 38.9 (C-4), 38.8 (C-1), 38.3 (C-13), 37.2 (C-22), 37.1 (C-10), 34.4 (C-7), 32.2 (C-16), 30.7 (C-21), 29.7 (C-15), 28.1(C-23), 27.5(C-2), 27.0 (CH_2_), 25.7 (CH_2_), 25.6 (C-21), 23.5 (CH_2_), 20.9 (C-11), 19.5 (C-30), 18.4 (C-6), 16.2 (C-26), 16.1 (C-25), 15.5 (C-24), 14.7 (C-27).

Compound **3k**: Yield: 73.9%; yellow colorless oil; IR (KBr, cm^-1^): ν_max_ 3488, 2916, 1695, 1632, 1034, 885. ^1^H NMR (CDCl_3_, 500 MHz) δ: 4.70 (1H, br s, Hb-29), 4.59 (1H, br s, Ha-29), 4.12 (2H, m, CH_2_), 3.68 (4H, t, *J* = 10 Hz, CH_2_), 3.15 (1H, dd, *J* = 4, 9.5 Hz, H-3a), 3.08 (1H, m, H-19), 2.26 (4H, m,CH_2_), 2.28 (2H, m, CH_2_), 1.67 (3H, s, H-30), 1.54 (m, CH_2_), 0.99 (6H, s, H-27), 0.92 (3H, s, H-23, H-26), 0.83 (3H, s, H-25), 0.73 (3H, s, H-24); ^13^C NMR (CDCl_3_, 125 MHz) δ: 176.3 (C-28), 150.7 (C-20), 109.6 (C-29), 78.9 (C-3), 63.9 (CH_2_), 59.1 (CH_2_), 56.6 (C-17), 55.4 (C-5), 54.6 (CH_2_), 50.6 (C-9), 49.4 (C-18), 47.1 (C-19), 42.5 (C-14), 40.8 (C-8), 38.9 (C-4), 38.8 (C-1), 38.3 (C-13), 37.2 (C-22), 37.1 (C-10), 34.4 (C-7), 32.2 (C-16), 30.7 (C-21), 29.7 (C-15), 28.1 (C-23), 27.5 (C-2), 27.0 (CH_2_), 25.6 (CH_2_), 25.6 (C-21), 24.5 (CH_2_), 23.5 (CH_2_), 20.9 (C-11), 19.5 (C-30), 18.4 (C-6), 16.2 (C-26), 16.1 (C-25), 15.5 (C-24), 14.7 (C-27).

Compound **3l**: Yield: 82.5%; yellow colorless oil; IR (KBr, cm^-1^): ν_max_ 3545, 2929, 2851, 1677, 1340, 887. ^1^H NMR (CDCl_3_, 500 MHz) δ: 4.69 (1H, br s, Hb-29), 4.59 (1H, br s, Ha-29), 4.11 (2H, m, CH_2_), 3.70(4H, t, *J* = 6.5 Hz, CH_2_), 3.15(1H, dd, J = 5.5, 11.5 Hz, H-3a), 2.96 (1H, m, H-19), 2.45 (4H, br s, CH_2_), 2.38 (2H, t, *J* = 7 Hz, CH_2_), 1.65 (3H, s, H-30), 1.58 (2H, m, CH_2_), 0.96 (6H, s, H-27), 0.87 (3H, s, H-23, H-26), 0.77 (3H, s, H-25), 0.72 (3H, s, H-24); ^13^C NMR (CDCl_3_, 125 MHz) δ: 176.2 (C-28), 150.6 (C-20), 109.7 (C-29), 78.9 (C-3), 66.7 (CH_2_), 63.7 (CH_2_), 58.4 (CH_2_), 56.6 (C-17), 55.4 (C-5), 53.5 (CH_2_), 50.6 (C-9), 49.4 (C-18), 47.1 (C-19), 42.5 (C-14), 40.8 (C-8), 38.9 (C-4), 38.8 (C-1), 38.3 (C-13), 37.2 (C-22), 37.1 (C-10), 34.4 (C-7), 32.2 (C-16), 30.7 (C-21), 29.7 (C-15), 28.1 (C-23), 27.5 (C-2), 26.7 (CH_2_), 25.6 (C-21), 22.8 (CH_2_), 20.9 (C-11), 19.4 (C-30), 18.4 (C-6), 16.2 (C-26), 16.1 (C-25), 15.5 (C-24), 14.8 (C-27).

***General procedure for compounds 5a-5f.***

Compound **2a** (1 mmol) and K_2_CO_3_ (2 mmol) were added to DMF (15 mL) and stirred at room temperature for 10 min, and then piperazine (5 mmol) was dripped into the mixture, which was stirred at 80 °C for 5 h. After cooling to room temperature, the reaction mixture was poured onto 100 mL of distilled water and partitioned with DCM (3×20 mL). The organic layer was washed with saturated sodium chloride, dried over Na_2_SO_4_ and purified via silica gel column chromatography with CHCl_3_/MeOH (10:1, v/v) to obtain compound **4**. Compound **4** (1 mmol), amine compounds (1.2 mmol), EDCI (1.2 mmol) and HOBt (1.2 mmol) were added to DCM (25 mL) containing Et_3_N (0.5 mmol); the mixture was then stirred at room temperature for 6–12 h. After the reaction was completed, the mixture was poured onto 100 mL of distilled water and partitioned with ethyl acetate (3×50 mL). The target compounds were purified on a flash column with chloroform/methanol (20:1, v/v) to yield compounds **5a-5f.**

Compound **5a**: Yield: 60.7%; colorless oil; IR (KBr, cm^-1^): ν_max_ 3451, 2926, 1679, 1361, 1044, 881, 711. ^1^H NMR (CDCl_3_, 500 MHz) δ: 7.38 (5H, s, PhH), 4.70 (1H, br s, Hb-29), 4.58 (1H, br s, Ha-29), 4.20 (2H, m, CH_2_), 3.77 (2H, s, H in piperazine), 3.40 (2H, s, H in piperazine), 3.16 (1H, dd, *J* = 5, 11.5 Hz, H-3a), 2.97 (1H, m, H-19), 2.65 (2H, t, *J* = 6 Hz, CH_2_), 2.59 (2H, br s, H in piperazine), 2.44 (2H, m, H in piperazine), 1.66 (3H, s, H-30), 0.99 (6H, s, H-27), 0.89 (3H, s, H-23, H-26), 0.77 (3H, s, H-25), 0.72 (3H, s, H-24); ^13^C-NMR (CDCl_3_, 125 MHz) δ: 176.0 (C-28), 170.4 (C), 150.6(C-20), 135.8(C), 129.7 (CH), 128.5 (CH), 127.1 (CH), 109.7 (C-29), 79.0 (C-3), 60.8 (CH_2_), 56.8 (CH_2_), 56.6 (C-17), 55.4 (C-5), 53.7 (CH_2_), 50.6 (C-9), 49.4 (C-18), 47.3(CH_2_), 47.0 (C-19), 42.5 (C-14), 40.8 (C-8), 38.9 (C-4), 38.8 (C-1), 38.3 (C-13), 37.2 (C-22), 37.1 (C-10), 34.4 (C-7), 32.2 (C-16), 30.6 (C-21), 29.7 (C-15), 28.0 (C-23), 27.5 (C-2), 25.6 (C-21), 20.9 (C-11), 19.4 (C-30), 18.4 (C-6), 16.2 (C-26), 16.1 (C-25), 15.5 (C-24), 14.8 (C-27).

Compound **5b**: Yield: 51.8%; colorless oil; IR (KBr, cm^-1^): ν_max_ 3446, 2931, 1682, 11641, 1012, 882. ^1^H NMR (CDCl_3_, 500 MHz) δ: 7.31 (2H, m, PhH), 4.69(1H, br s, Hb-29), 4.57 (1H, br s, Ha-29), 4.20 (2H, m, CH_2_), 3.81 (2H, m, H in piperazine), 3.21(2H, t, *J* = 6.5 Hz, H in piperazine), 3.16(1H, dd, *J* = 4, 8.5 Hz, H-3a), 2.97 (1H, m, H-19), 2.65 (2H, t, *J* = 4.5 Hz, CH_2_), 2.59 (2H, t, *J* = 5 Hz, H in piperazine), 2.48 (2H, m, H in piperazine), 1.65 (3H, s, H-30), 0.97 (6H, s, H-27), 0.88 (3H, s, H-23, H-26), 0.77 (3H, s, H-25), 0.72 (3H, s, H-24); ^13^C NMR (CDCl_3_, 125 MHz) δ: 176.0 (C-28), 163.7 (C), 150.6 (C-20), 135.0 (C), 131.8 (C), 130.4 (CH), 128.1 (CH), 109.7 (C-29), 79.3 (C-3), 60.8 (CH_2_), 56.7 (CH_2_), 56.6 (C-17), 55.4 (C-5), 53.3 (CH_2_), 52.7 (CH_2_), 50.6 (C-9), 49.4 (C-18), 47.0 (C-19), 46.3 (CH_2_), 42.5 (C-14), 41.6 (CH_2_), 40.8 (C-8), 38.9 (C-4), 38.8 (C-1), 38.3 (C-13), 37.2 (C-22), 37.1 (C-10), 34.4 (C-7), 32.2 (C-16), 30.6 (C-21), 29.7 (C-15), 28.0 (C-23), 27.5 (C-2), 25.6 (C-21), 20.9 (C-11), 19.4 (C-30), 18.4 (C-6), 16.2 (C-26), 16.1 (C-25), 15.5 (C-24), 14.8 (C-27).

Compound **5c**: Yield: 56.9%; yellow colorless oil; IR (KBr, cm^-1^): ν_max_ 3477, 2918, 2867, 1680, 1635, 1040, 883, 743. ^1^H NMR (CDCl_3_, 500 MHz) δ: 7.29 (2H, m, PhH), 7.05(1H, t, *J* = 7 Hz, PhH), 4.70 (1H, br s, Hb-29), 4.57 (1H, br s, Ha-29), 4.20 (2H, m, CH_2_), 3.82 (2H, t, *J* = 5.5 Hz, H in piperazine), 3.26 (2H, t, *J* = 3.5 Hz, H in piperazine), 3.16(1H, dd, *J* = 4.5, 9 Hz, H-3a), 2.97 (1H, m, H-19), 2.65 (2H, t, *J* = 4.5 Hz, CH_2_), 2.59 (2H, t, J = 6 Hz, H in piperazine), 2.50 (2H, m, H in piperazine), 1.68 (3H, s, H-30), 0.95 (6H, s, H-27), 0.88 (3H, s, H-23, H-26), 0.78 (3H, s, H-25), 0.73 (3H, s, H-24); ^13^C NMR (CDCl_3_, 125 MHz) δ: 176.0 (C-28), 161.9 (C), 159.7 (C), 157.7 (C), 150.7 (C-20), 131.9 (CH), 130.8 (CH), 125.6 (CH), 114.6 (CH), 109.7 (C-29), 79.3 (C-3), 60.8 (CH_2_), 56.7 (CH_2_), 56.6 (C-17), 55.4 (C-5), 53.4 (CH_2_), 50.6 (C-9), 49.4 (C-18), 47.0 (C-19), 46.6 (CH_2_), 42.5 (C-14), 41.8 (CH_2_), 40.8 (C-8), 38.9 (C-4), 38.8 (C-1), 38.3 (C-13), 37.2 (C-22), 37.1 (C-10), 34.4 (C-7), 32.2 (C-16), 30.6 (C-21), 29.7 (C-15), 28.0 (C-23), 27.5 (C-2), 25.6 (C-21), 20.9 (C-11), 19.4 (C-30), 18.3 (C-6), 16.2 (C-26), 16.1 (C-25), 15.5 (C-24), 14.8 (C-27).

Compound **5d**: Yield: 55.5%; yellow colorless oil; IR (KBr, cm^-1^): ν_max_ 3451, 2934, 2860, 1689, 1643, 1049, 885, 763. H NMR (CDCl_3_ 500 MHz) δ: 7.31 (2H, d, *J* = 6 Hz, PhH), 7.24 (2H, d, *J* = 7 Hz, PhH), 4.70 (1H, br s, Hb-29), 4.58 (1H, br s, Ha-29), 4.22 (2H, m, CH_2_), 3.75 (1H, br s, H in piperazine), 3.35(1H, dd, *J* = 4, 10 Hz, H-3a), 2.97 (1H, m, H-19), 2.64 (2H, t, *J* = 7.5 Hz, CH_2_), 1.67 (3H, s, H-30), 0.97 (6H, s, H-27), 0.89 (3H, s, H-23, H-26), 0.78 (3H, s, H-25), 0.72 (3H, s, H-24); ^13^C NMR (CDCl_3_, 125 MHz) δ: 176.0 (C-28), 170.5 (C=O), 150.8 (C-20), 141.9 (C), 133.2 (C), 127.3 (CH), 126.5 (CH), 109.7 (C-29), 79.0 (C-3), 60.9 (CH_2_), 56.8 (CH_2_), 56.6 (C-17), 55.4 (C-5), 50.6 (C-9), 49.4 (C-18), 47.0 (C-19), 42.5 (C-14), 40.8 (C-8), 38.9 (C-4), 38.8 (C-1), 38.3 (C-13), 37.2 (C-22), 37.1 (C-10), 34.4 (C-7), 32.2 (C-16), 30.6 (C-21), 29.7 (C-15), 28.0 (C-23), 27.5 (C-2), 25.6 (C-21), 23.9 (CH_3_), 20.9 (C-11), 19.4 (C-30), 18.4 (C-6), 16.2 (C-26), 16.1 (C-25), 15.5 (C-24), 14.8 (C-27).

Compound **5e**: Yield: 62.8%; yellow colorless oil; IR (KBr, cm^-1^): ν_max_ 3450, 2920, 2861, 1683, 1645, 1377, 1044, 885,743. ^1^H NMR (CDCl_3_, 500 MHz) δ: 7.31 (2H, m, PhH), 7.11 (3H, m, PhH), 4.70 (1H, br s, Hb-29), 4.58 (1H, br s, Ha-29), 4.20 (2H, m, CH_2_), 3.77 (2H, br s, H in piperazine), 3.40 (2H, br s, H in piperazine), 3.16 (1H, dd, *J* = 6, 11.5 Hz, H-3a), 2.97 (1H, m, H-19), 2.65 (2H, t, *J* = 5 Hz, CH_2_), 2.59 (2H, t, *J* = 4.5 Hz, H in piperazine), 2.45 (2H, m, H in piperazine), 1.66 (3H, s, H-30), 0.96 (6H, s, H-27), 0.89 (3H, s, H-23, H-26), 0.79 (3H, s, H-25), 0.73 (3H, s, H-24); ^13^C NMR (CDCl_3_, 125 MHz) δ: 176.0 (C-28), 168.8 (C=O), 161.6 (C), 150.6 (C-20), 137.8 (CH), 130.4 (CH), 122.2 (CH), 116.9 (CH), 114.5 (CH), 109.7 (C-29), 79.3 (C-3), 60.8 (CH_2_), 56.7 (CH_2_), 56.6 (C-17), 55.4 (C-5), 53.6 (CH_2_), 50.6 (C-9), 49.4 (C-18), 47.0 (C-19), 46.6 (CH_2_), 42.5 (C-14), 42.2 (CH_2_), 40.8 (C-8), 38.9 (C-4), 38.8 (C-1), 38.3 (C-13), 37.2 (C-22), 37.1 (C-10), 34.4 (C-7), 32.2 (C-16), 30.6 (C-21), 29.7 (C-15), 28.0 (C-23), 27.5 (C-2), 25.6 (C-21), 20.9 (C-11), 19.4 (C-30), 18.4 (C-6), 16.2 (C-26), 16.1 (C-25), 15.5 (C-24), 14.8 (C-27).

Compound **5f**: Yield: 60.8%; yellow colorless oil; IR (KBr, cm^-1^): ν_max_ 3456, 2926, 2867, 1681, 1640, 1039, 886,732. ^1^H NMR (CDCl_3_, 500 MHz) δ: 7.66 (1H, d, *J* = 15.5 Hz, PhH), 7.50(2H, d, *J* = 4.5 Hz, PhH), 7.35 (2H, m, PhH), 6.86 (1H, d, *J* = 14 Hz, CH), 4.70 (1H, br s, Hb-29), 4.57 (1H, br s, Ha-29), 4.22 (2H, m, CH_2_), 3.71 (2H, s, H in piperazine), 3.40 (2H, s, H in piperazine), 3.15 (1H, dd, *J* = 5, 11.5 Hz, H-3a), 2.97 (1H, m, H-19), 2.64 (2H, t, *J* = 8 Hz, CH_2_), 2.52 (4H, t, *J* = 5 Hz, H in piperazine), 1.65 (3H, s, H-30), 0.98 (6H, s, H-27), 0.89 (3H, s, H-23, H-26), 0.79 (3H, s, H-25), 0.72 (3H, s, H-24); ^13^C NMR (CDCl_3_, 125 MHz) δ: 176.0 (C-28), 165.5 (C), 150.6 (C-20), 142.9 (CH), 135.3 (C), 129.7 (CH), 128.8 (CH), 127.8 (CH), 117.1 (CH), 109.8 (C-29), 79.0 (C-3), 60.8 (CH_2_), 56.8 (CH_2_), 56.6 (C-17), 55.4 (C-5), 53.7 (CH_2_), 50.6 (C-9), 49.4 (C-18), 47.0 (C-19), 42.5 (C-14), 40.8 (C-8), 38.9 (C-4), 38.8 (C-1), 38.3 (C-13), 37.2 (C-22), 37.1 (C-10), 34.4 (C-7), 32.2 (C-16), 30.6 (C-21), 29.7 (C-15), 28.0 (C-23), 27.5 (C-2), 25.6 (C-21), 20.9 (C-11), 19.4 (C-30), 18.4 (C-6), 16.2 (C-26), 16.1 (C-25), 15.5 (C-24), 14.8 (C-27).

## Copies of 1H-NMR and 13C-NMR of selected compounds


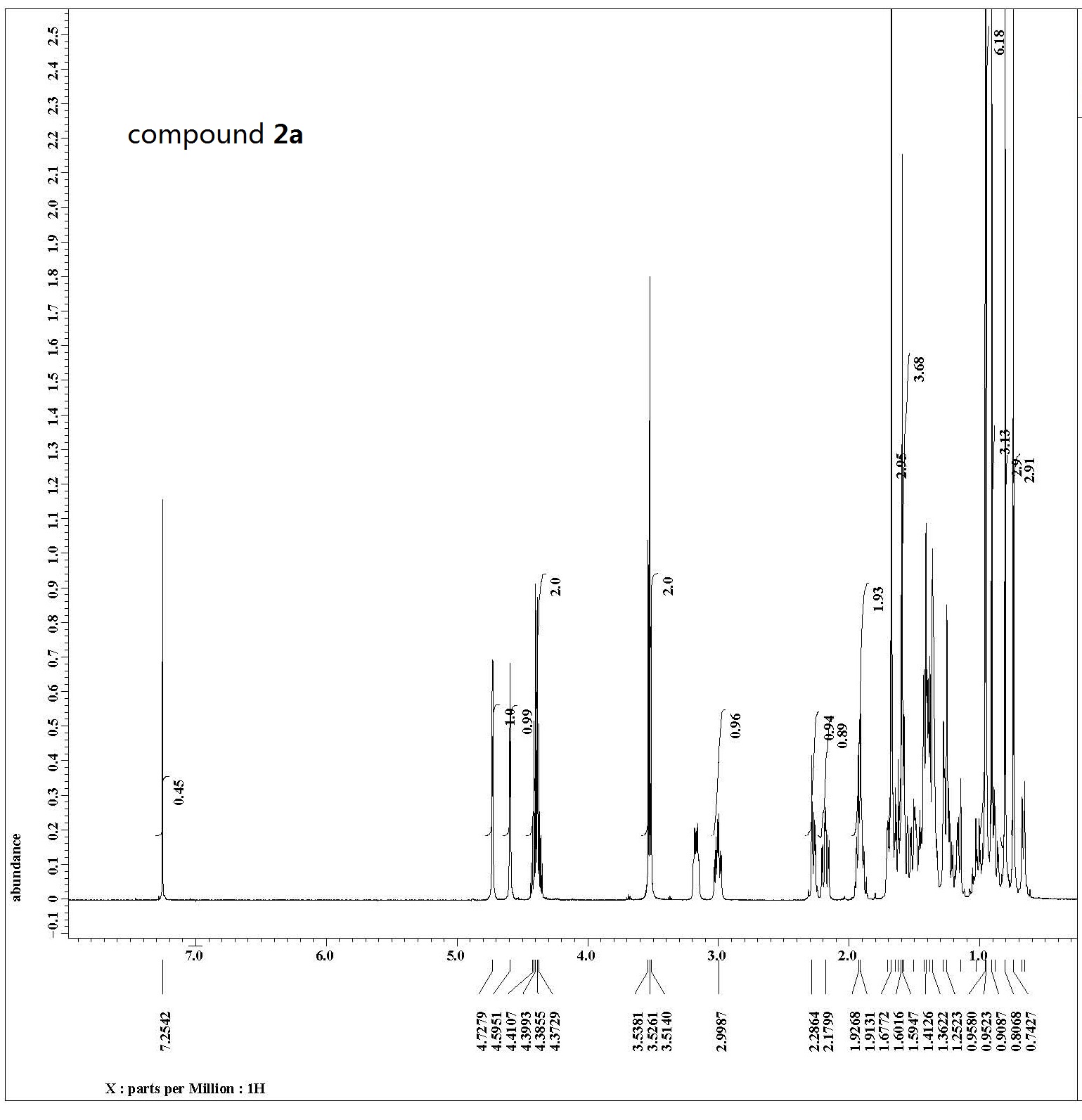


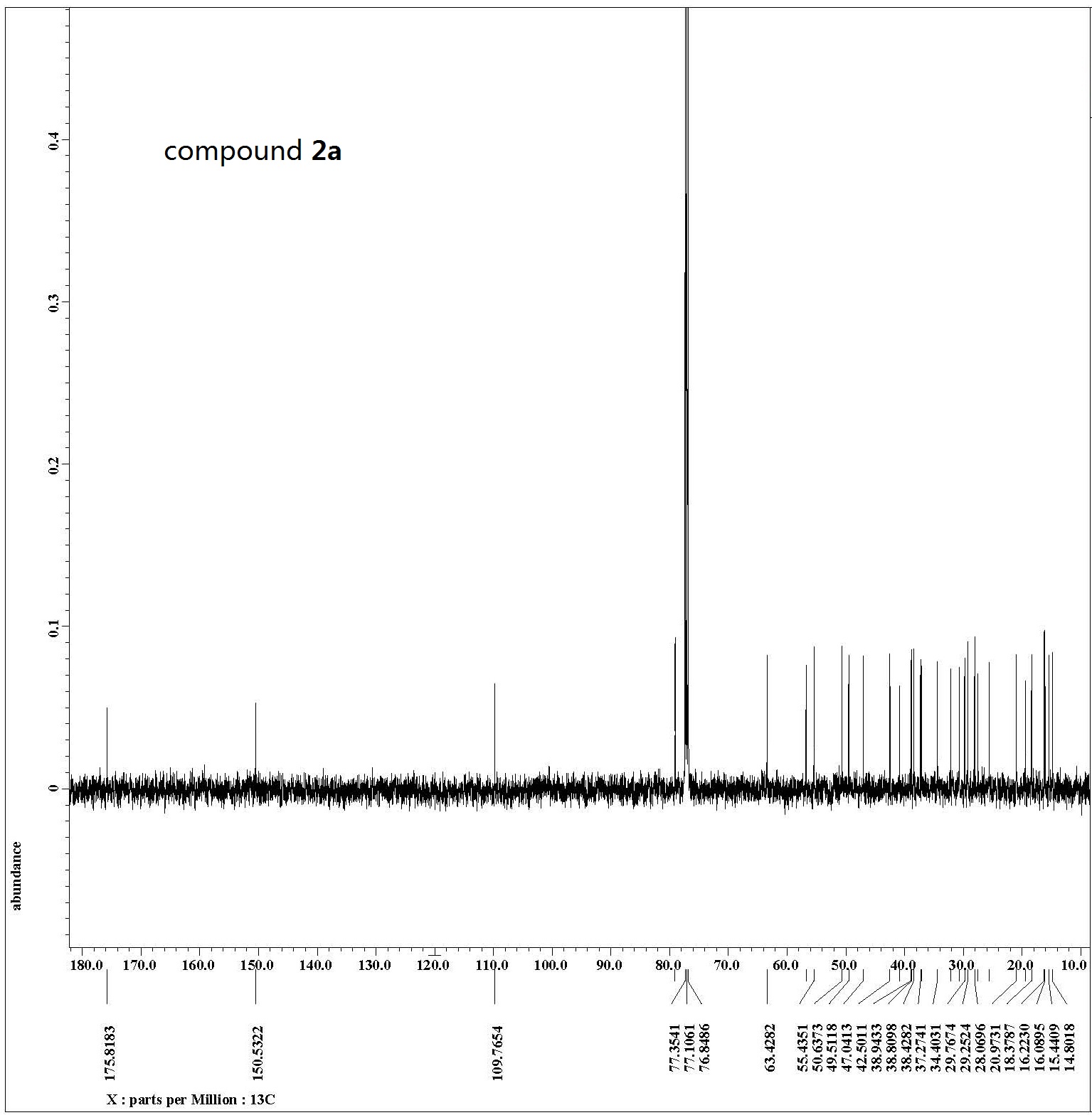


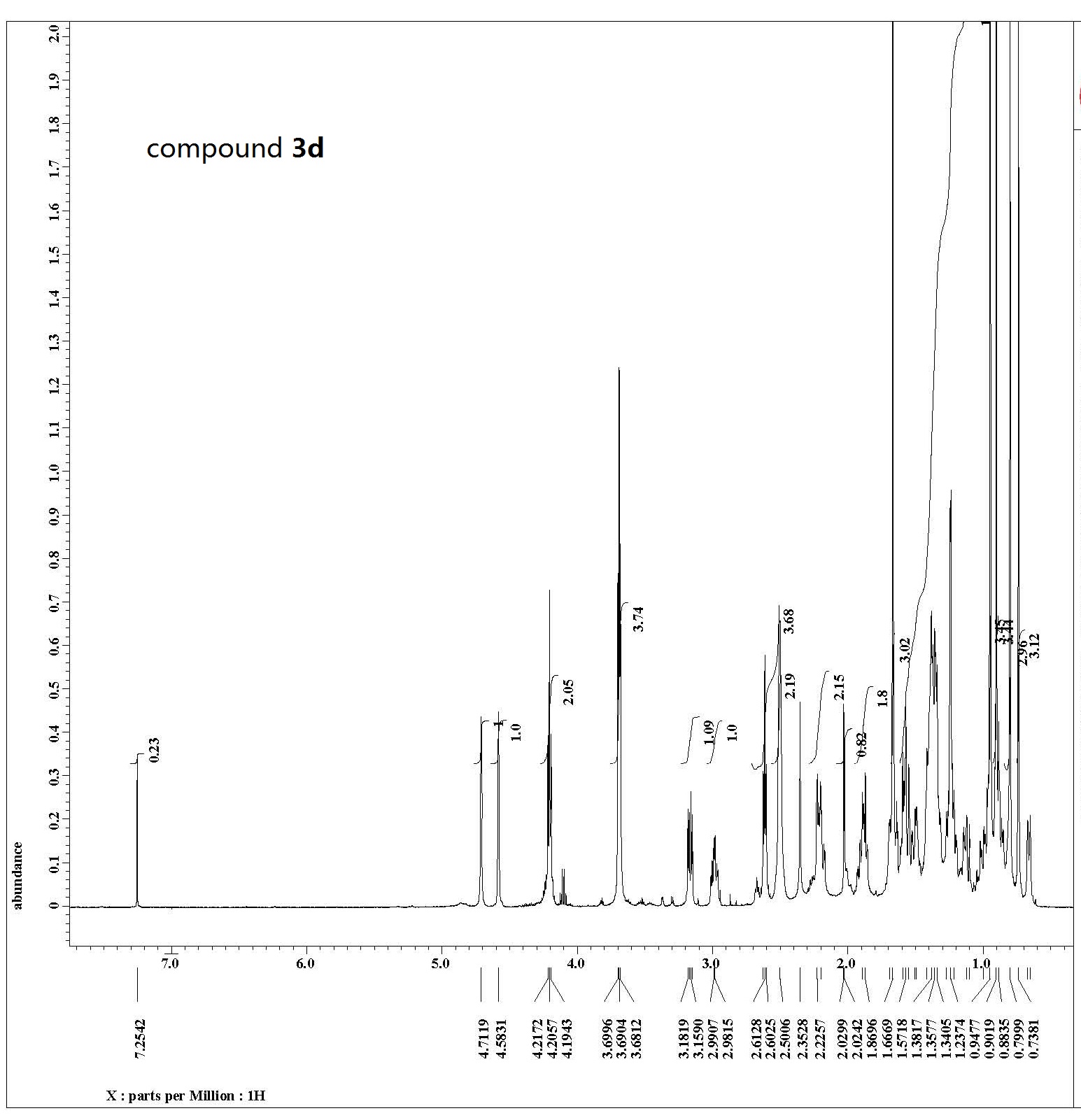


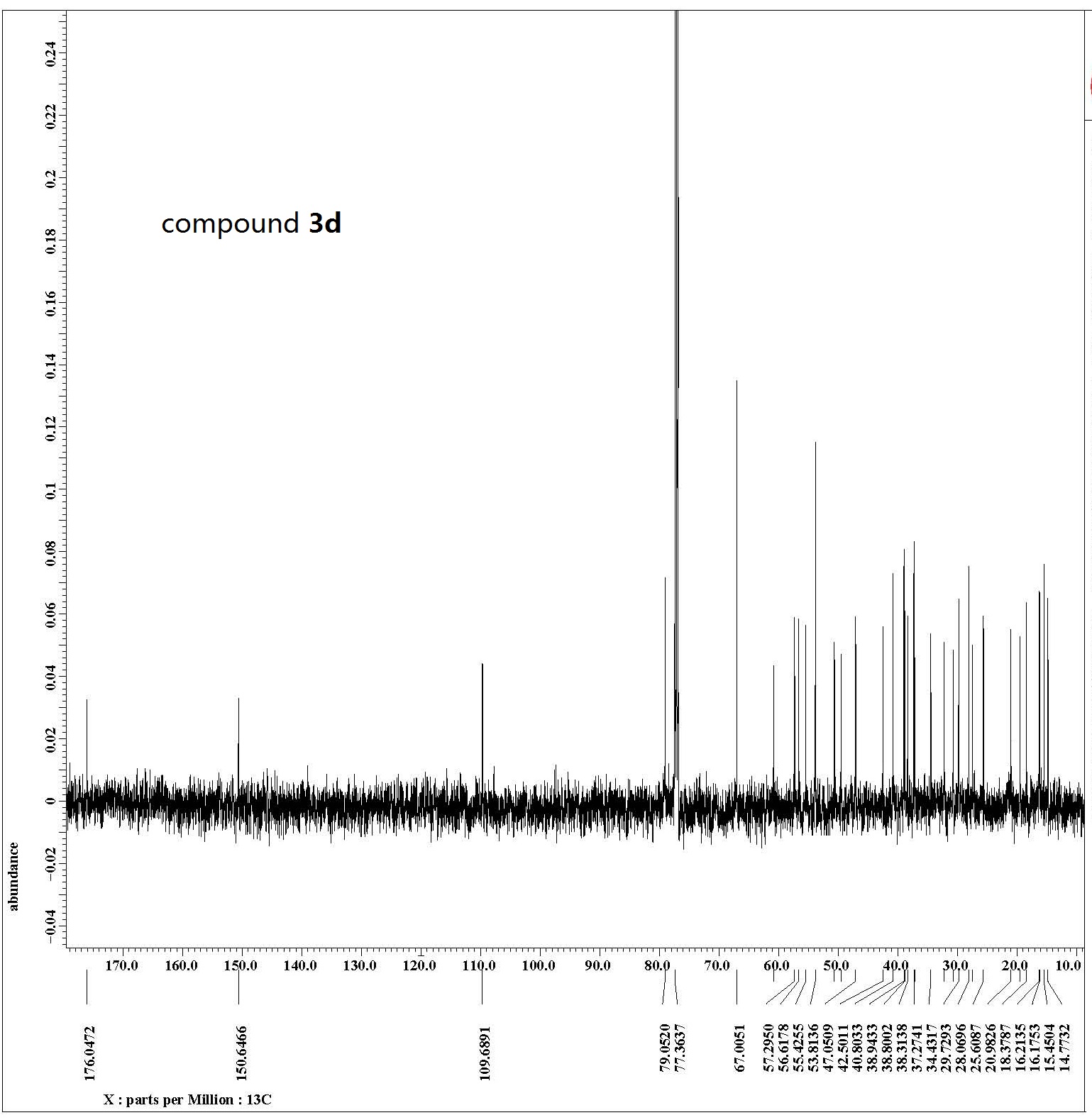


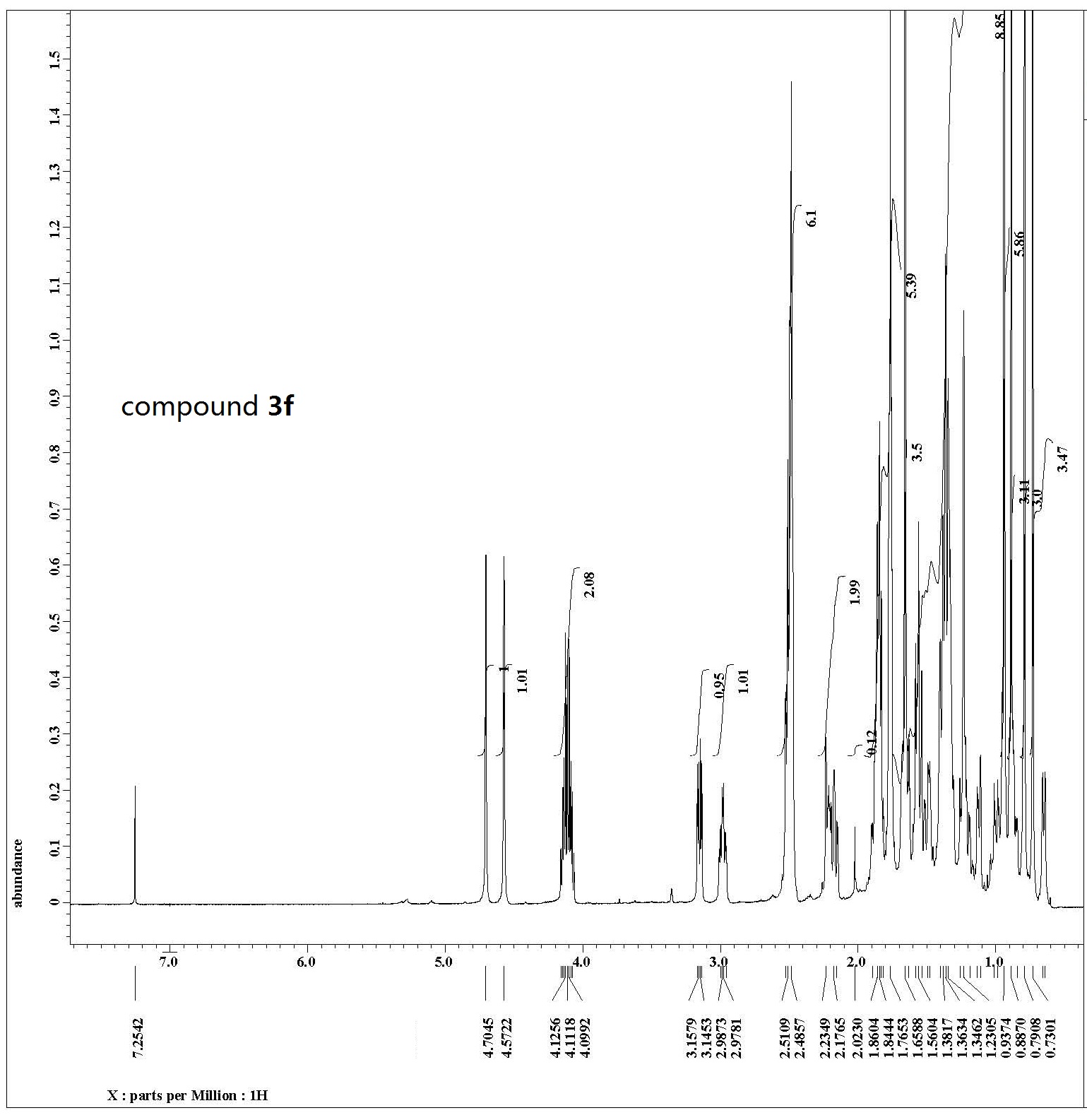


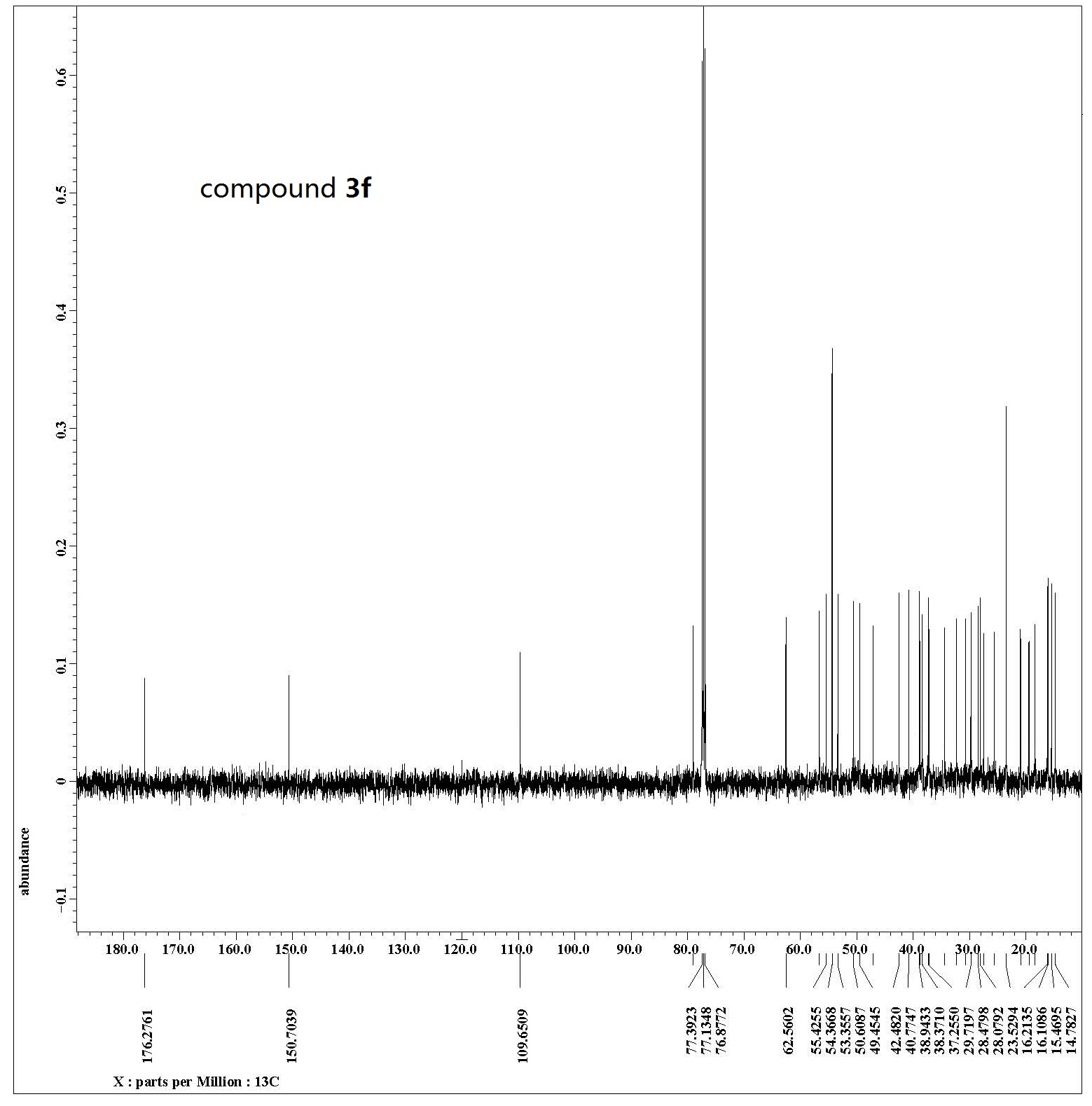


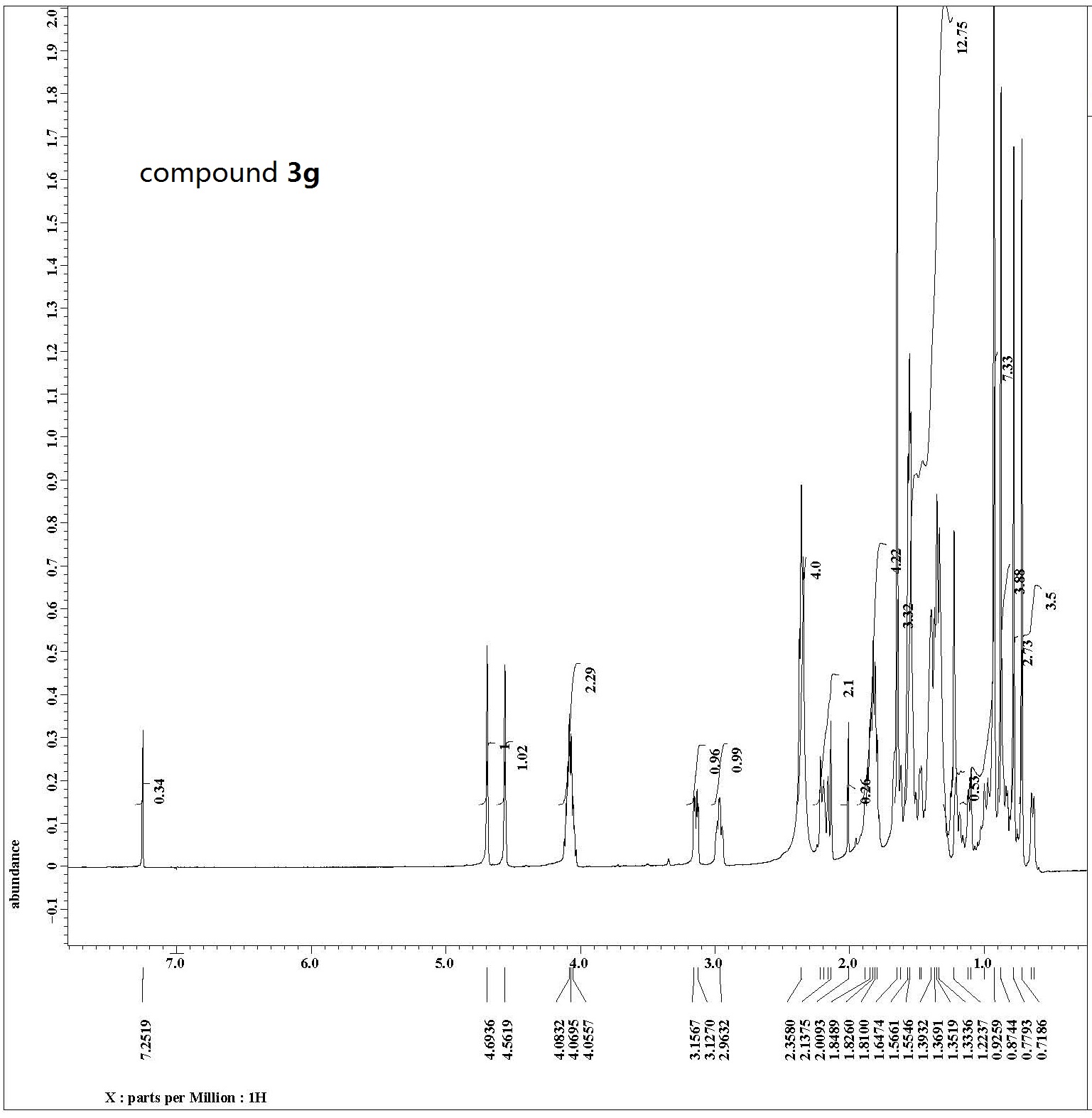


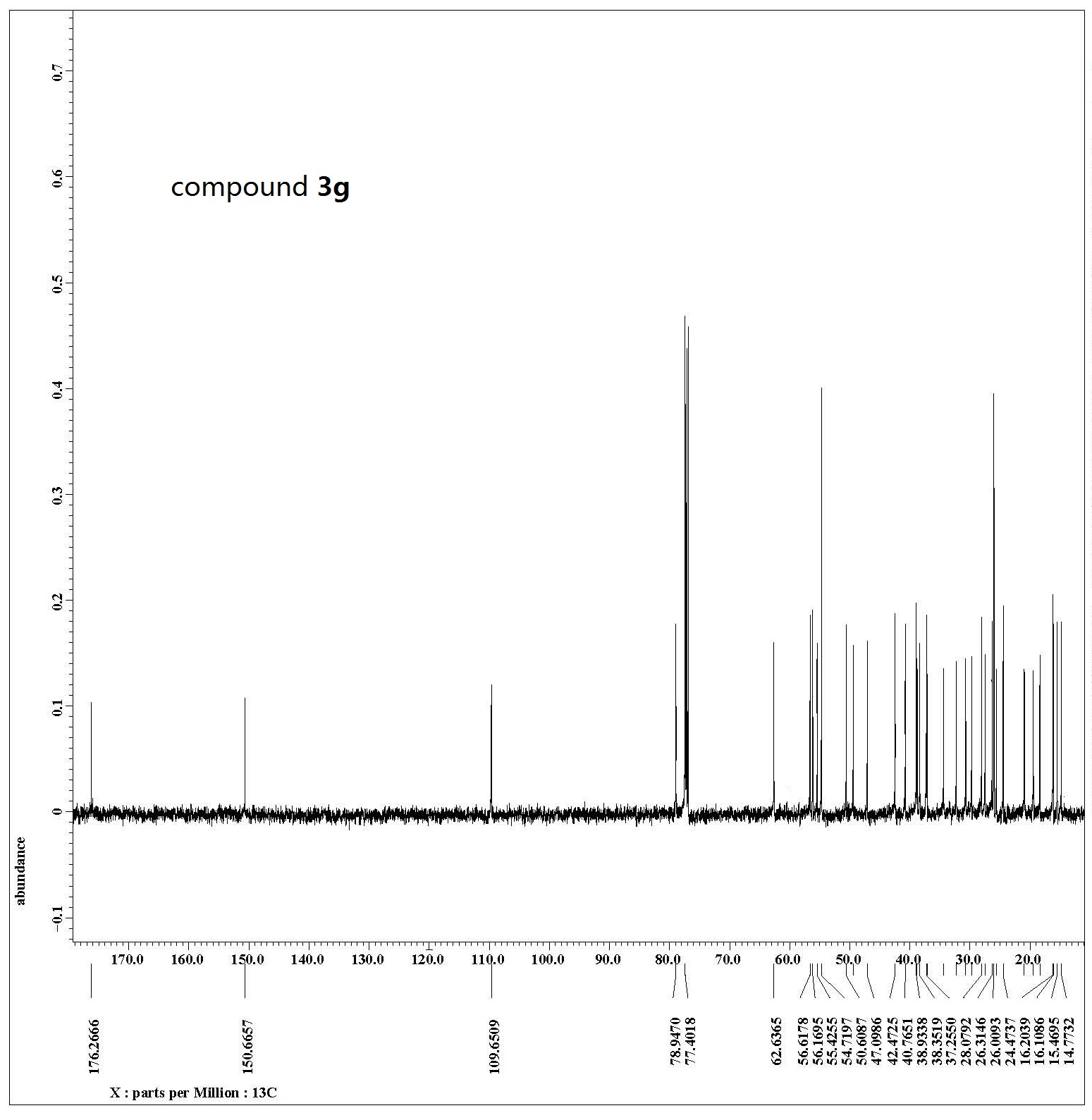


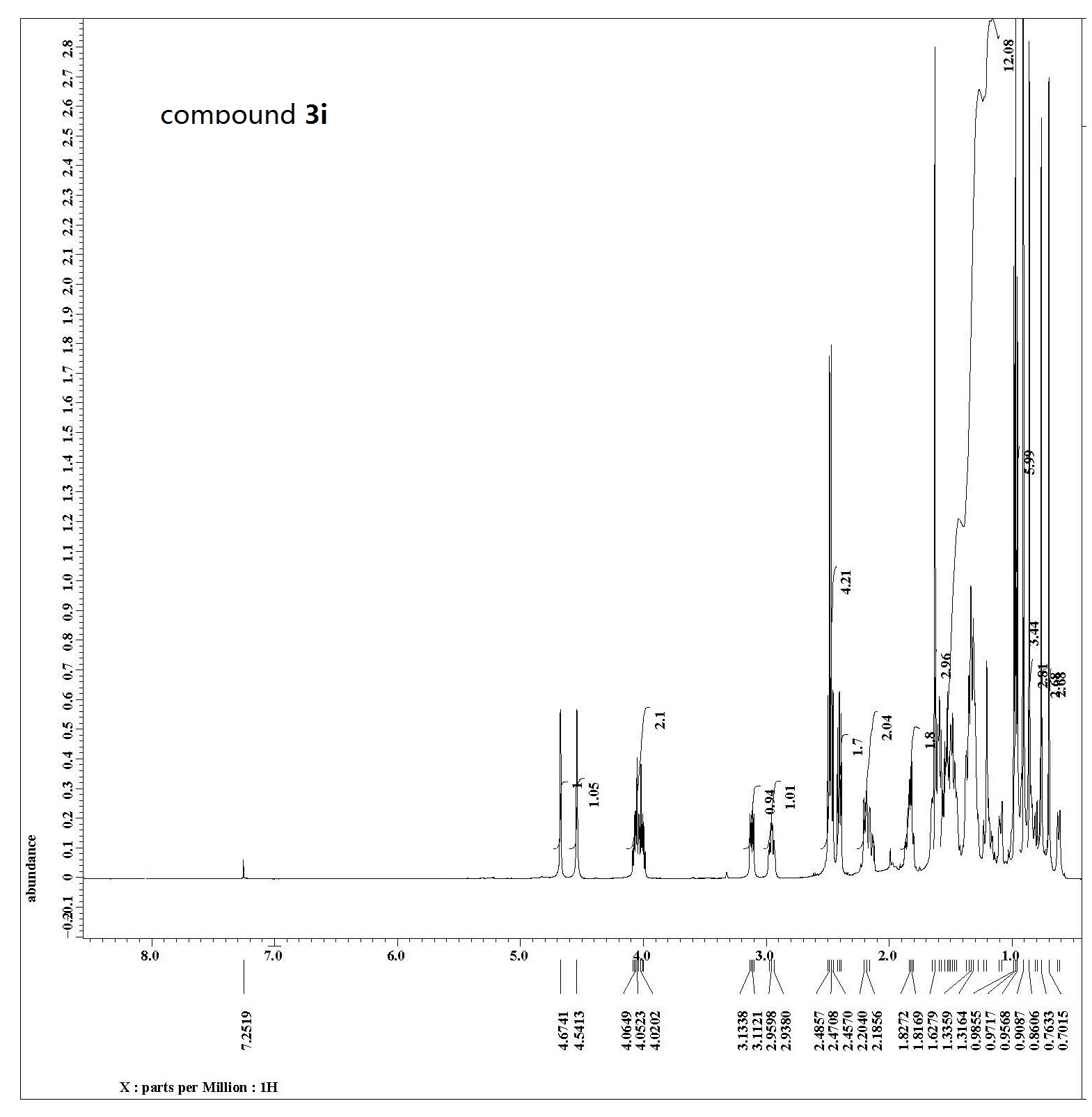


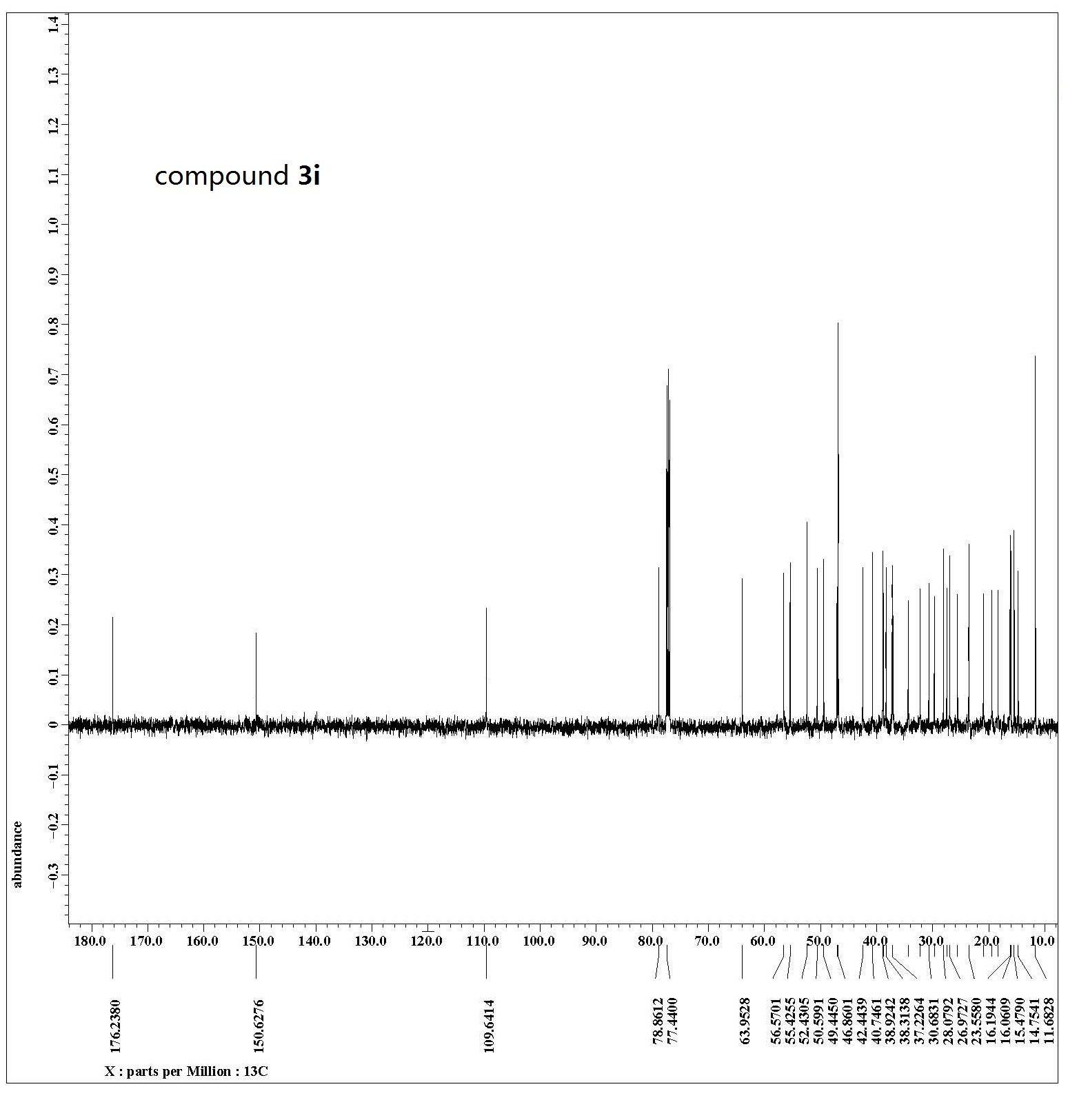


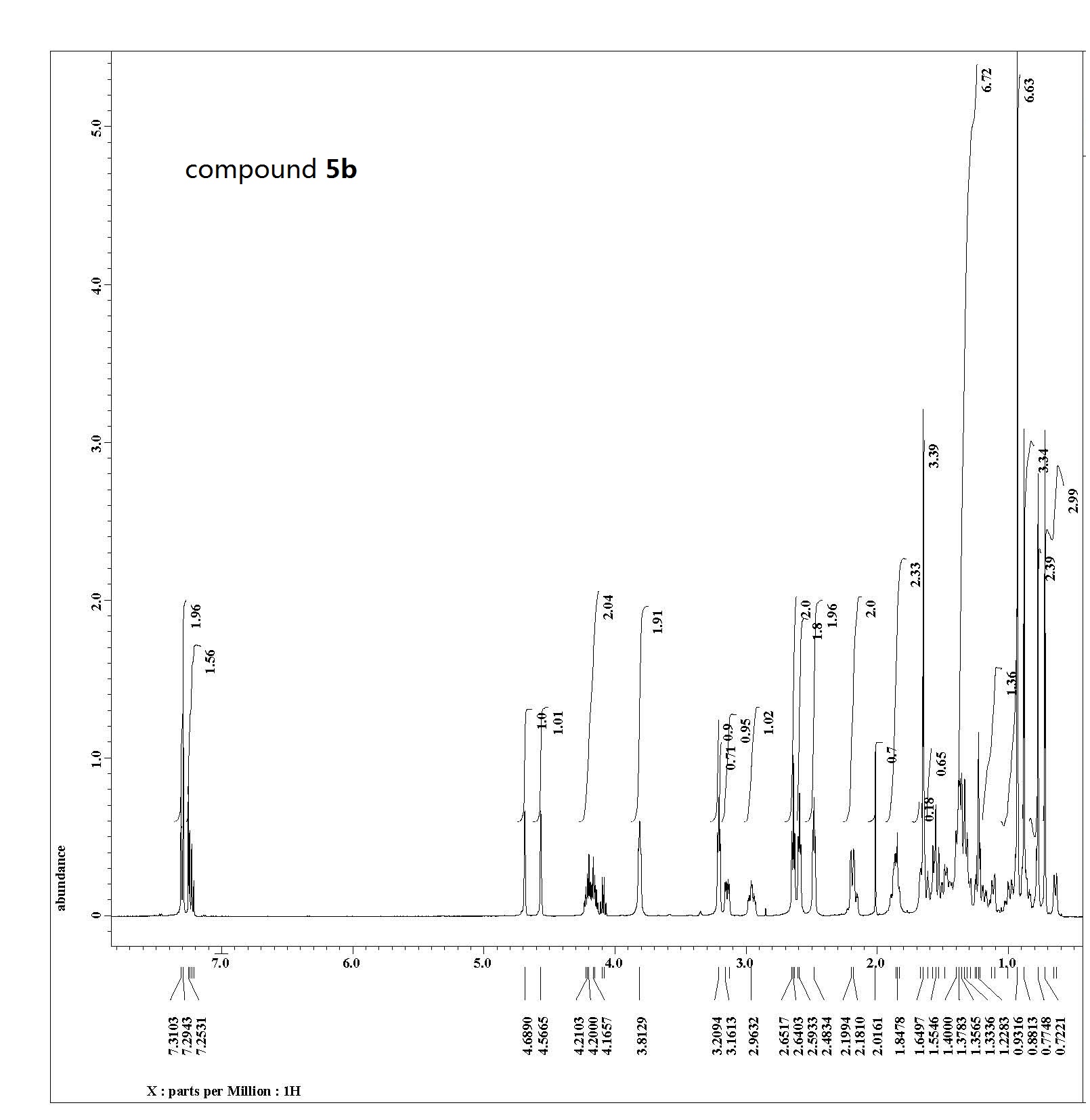


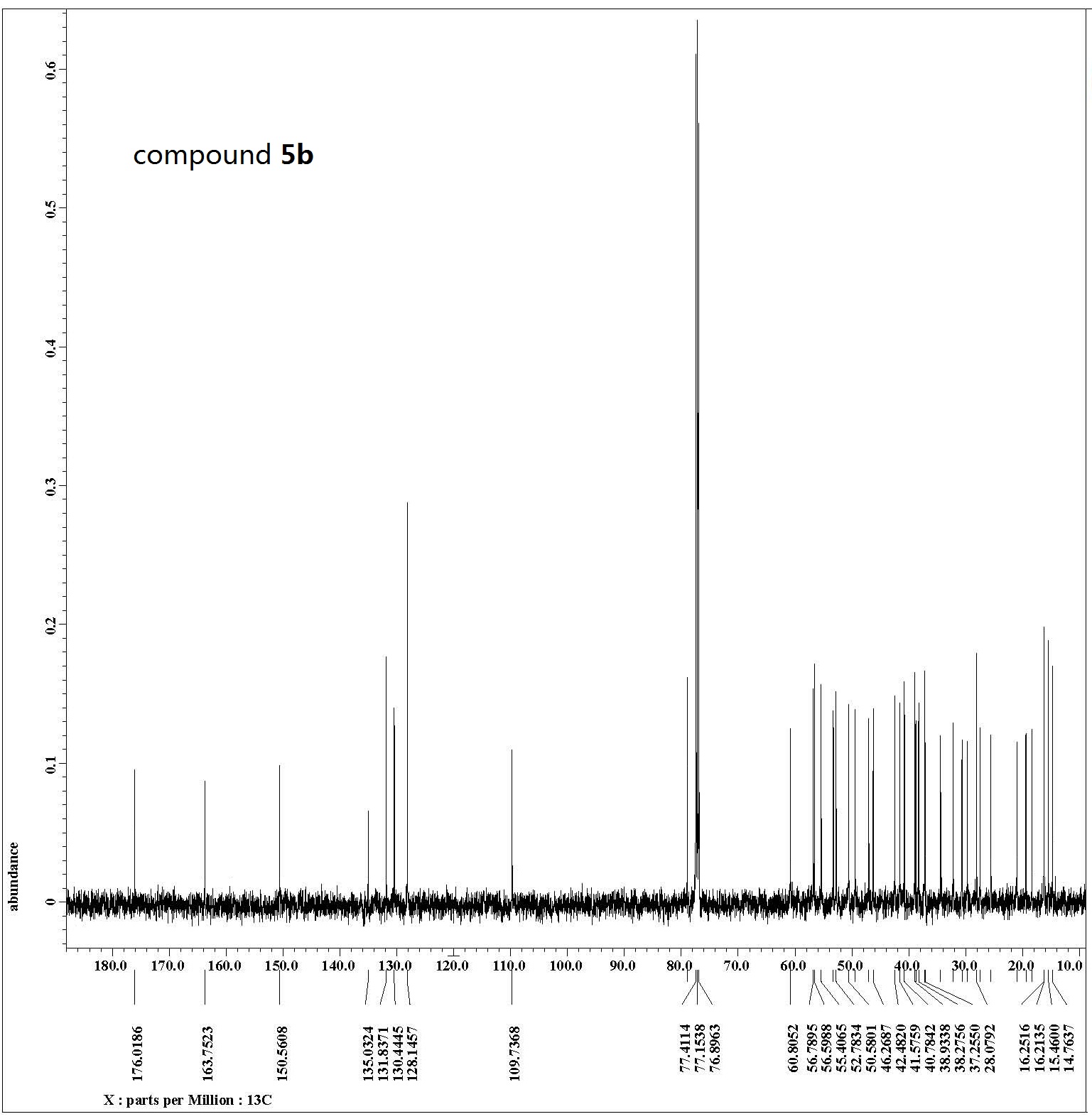

Supplement: Additional file 1 — Experimental details and data of BA derivatives. Which includes the experimental procedure, spectroscopic data, and copies of 1H NMR and 13C NMR of selected compounds. [file 1752-153X-6-141-S1.doc]
